# Local adaptations in bryophytes revisited: the genetic structure of the calcium-tolerant peatmoss *Sphagnum warnstorfii* along geographic and pH gradients

**DOI:** 10.1002/ece3.1351

**Published:** 2014-12-19

**Authors:** Eva Mikulášková, Michal Hájek, Adam Veleba, Matthew G Johnson, Tomáš Hájek, Jonathan A Shaw

**Affiliations:** 1 Department of Botany and Zoology, Faculty of Science, Masaryk UniversityKotlářská 2, Brno, CZ 61137, Czech Republic; 2 Department of Vegetation Ecology, Institute of Botany, Academy of Sciences of the Czech RepublicLidická 25/27, Brno, CZ 65720, Czech Republic; 3 Plant Science and Conservation, Chicago Botanic Gardens1000 Lake Cook Road, Glencoe, IL, 60022, USA; 4 Department of Functional Ecology, Institute of Botany, Academy of Sciences of the Czech RepublicDukelská 135, Třeboň, CZ 37982, Czech Republic; 5 Department of Biology, Duke UniversityDurham, NC, 27708, USA

**Keywords:** Calcium tolerance, ecotypic adaptation, hybridization, microsatellites, population structure, *Sphagnum warnstorfii*

## Abstract

Bryophytes dominate some ecosystems despite their extraordinary sensitivity to habitat quality. Nevertheless, some species behave differently across various regions. The existence of local adaptations is questioned by a high dispersal ability, which is thought to redistribute genetic variability among populations. Although *Sphagnum warnstorfii* is an important ecosystem engineer in fen peatlands, the causes of its rather wide niche along the pH/calcium gradient are poorly understood. Here, we studied the genetic variability of its global populations, with a detailed focus on the wide pH/calcium gradient in Central Europe. Principal coordinates analysis of 12 polymorphic microsatellite loci revealed a significant gradient coinciding with water pH, but independent of geography; even samples from the same fens were clearly separated along this gradient. However, most of the genetic variations remained unexplained, possibly because of the introgression from phylogenetically allied species. This explanation is supported by the small heterogeneous cluster of samples that appeared when populations morphologically transitional to *S. subnites, S. rubellum*, or *S. russowii* were included into the analysis. Alternatively, this unexplained variation might be attributed to a legacy of glacial refugia with recently dissolved ecological and biogeographic consequences. Isolation by distance appeared at the smallest scale only (up to 43 km). Negative spatial correlations occurred more frequently, mainly at long distances (up to 950 km), implying a genetic similarity among samples which are very distant geographically. Our results confirm the high dispersal ability of peatmosses, but simultaneously suggested that their ability to cope with a high pH/calcium level is at least partially determined genetically, perhaps via specific physiological mechanisms or a hummock-forming ability.

## Introduction

During the last few decades, advances in molecular genetic techniques have allowed more in-depth inquiry into long-standing questions in ecology and biogeography. One of these is the spatial instability of species’ realized niches observed in nature, as determined by the so-called ecotypes – ecologically different genotypes within a species resulting from an adaptation to local environmental conditions (Turesson 1922; Hufford and Mazer 2003). There is increasing evidence that the genetic patterns associated with local genetic adaptation are highly important (Lee and Mitchell-Olds 2011) and they are as common as the hitherto predominantly emphasized patterns emerging from isolation by dispersal limitation (Orsini et al. 2013). Focusing on the local adaptations within particular species may thus help to understand the distribution patterns observed in nature. Nevertheless, little is known about local adaptations in spore-producing plants such as bryophytes, where a high dispersal ability (Hájek et al. 2011; Sundberg 2013) continuously redistributes genetic variability among distant populations (Szövényi et al. 2008; Vanderpoorten et al. 2008) and hence counteracts local adaptation. There are few studies testing the coincidence between the variation in genetic structure and environmental factors in bryophytes. While Karlin et al. (2011) found that all plants of *Sphagnum subnitens* distributed from the Pacific coast of the continental United States (Oregon) to the western end of the Aleutian Islands appear to comprise a single genotype, some other studies on peatmoss genetics found at least a small signal of the effect of the local environment on genetic structure (Såstad et al. 1999; Gunnarsson et al. 2007; Hutsemekers et al. 2010). In addition, Szövényi et al. (2009) found certain indications of local adaptations within the extremely well-dispersing peatmoss *Sphagnum fimbriatum* using an analysis of a spatial pattern in a nucleotide polymorphism. These studies encourage us to search for further evidence of local adaptations, even within a group of well-dispersed organisms. In this study, we applied the concept of local adaptation (i.e., isolation by the environment in the sense of Orsini et al. 2013) into the long-standing question of the ecological tolerances of calcium-tolerant peatmosses, which act as important ecosystem engineers in fens.

**Figure 1:**
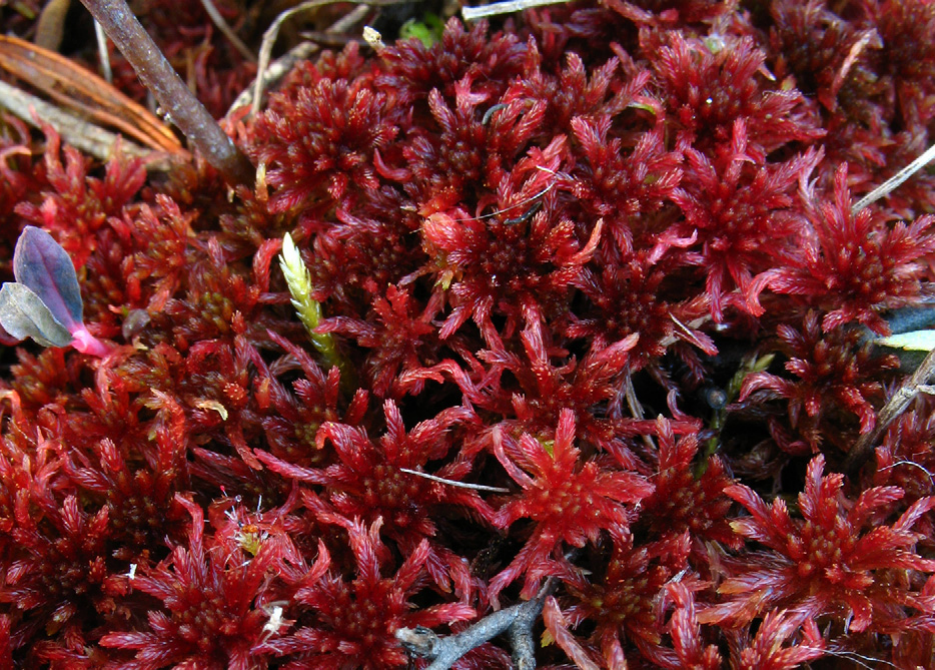
*Sphagnum warnstorfii*, the model organism of our study.

Studies exploring taxonomic diversity in fens have demonstrated that pH and calcium are leading factors determining the species composition and the diversity of fens throughout the world (see, Hájek et al. 2006 for review). For peatmosses (*Sphagnum* L.) and other bryophytes, interspecific differences in the tolerance to superfluous calcium in tissues are a major explanation of this strong compositional gradient (Hájek et al. 2014); under high calcium uptake, cells have to maintain very low cytoplasmic concentrations of calcium by excluding it to apoplast. Although calcium oversupply is a causal explanation of the pattern, with the most tolerant peatmoss species being named *calcium-tolerant*, the distribution of a particular peatmoss species is best explained by the water pH in our study areas (Z. Plesková, M. Hájek, T. Peterka, P. Hájková, D. Dítč, unpubl. data). It is because the calcium uptake is increasing with increasing pH; a high calcium concentration does not matter under a low pH, and vice versa. In addition, in our study areas, the same geochemical process determines high pH and a high calcium level via the enrichment of the groundwater by calcium hydrogencarbonate. *Sphagnum warnstorfii* (Fig.1) is the most prominent calcium-tolerant species, frequently occurring and even dominating in rich fens. It is therefore a suitable model organism to test whether the tolerance of the peatmoss species to calcium differs within species and whether it is determined genetically. In Central Europe, *S. warnstorfii* predominantly grows on rich fens in a slightly acid to neutral pH (See Table1 for details). Some authors have described different pH ranges for *S. warnstorfii* and other peatmoss species in different regions, suggesting a shift in the species’ niche (Dierssen and Dierssen 2001; Hájková and Hájek 2007; Hájková et al. 2008; see also Table1). Pakarinen (1979) hypothesized that this broad occurrence may result from the existence of genetically differentiated ecotypes within *S. warnstorfii*. This pattern is similar to the variable habitat affinities of many fen species observed in different regions. Hájková et al. (2008) speculated that the evolution of calcium-level ecotypes may have occurred during the Pleistocene, in refugial populations occurring in calcium-rich versus calcium-poor habitats. This explanation, however, might apply especially to vascular plants experiencing higher levels of relictualism caused by longer life spans and worse dispersal abilities as compared to bryophytes (Horsák et al. 2012). The existence of isolation by environment within the model bryophyte species may therefore be more fruitful. We are not aware of any study that would test the pattern in the genetic differentiation of plants along the sufficiently long pH/calcium gradient at the landscape scale. The studies on acidicole peatmosses (*Sphagnum fuscum* – Gunnarsson et al. 2007; *Sphagnum angustifolium –* Såstad et al. 1999) involved a rather short pH gradient, and their results cannot therefore be extrapolated to our question of different levels of calcium tolerance.

**Table 1:** Selected literature reports on pH optima of *Sphagnum warnstorfii* from different regions, illustrating its wide pH niche and its tolerance to rather high pH values

Region	pH min.	pH opt.	pH max.
Northern Minnesota (Vitt and Slack 1984)	n/a	7.4	n/a
Schwarzwald (Dierssen and Dierssen 1984)	4.2	4.9	5.5
Canada (Andrus 1986)	4.0	6.2	7.5
Maine, Northern America (Anderson et al. 1995)	n/a	8.3	n/a
Western Italian Alps (Miserere et al. 2003)	n/a	5.4	n/a
Central Europe (Hájek et al. 2006)	5.8	n/a	6.9
Bulgaria (Hájková and Hájek 2007)*	4.3	5.4	6.1
Poland, Sudety Mts. (Wojtuń et al. 2013)	n/a	5.4	n/a
Western Carpathians (Z. Plesková et al., unpubl. data)	3.8	6.0	7.6
Bohemian Massif (Z. Plesková et al., unpubl. data)	3.7	6.2	7.6

pH min/pH max, minimal pH/maximal pH; pH opt., pH optimum (highest peak of the species response curve, mean value or median value; the methodology differed among studies).

* Minimum, maximum, and median were recalculated from the original dataset.

Our study is hence focused on the calcium-tolerant species *Sphagnum warnstorfii*, which sometimes tolerates very high pH and calcium levels around mineral-rich springs, but sometimes it avoids even slightly calcium-enriched fens (Hájková and Hájek 2007). This phenomenon could be explained by genetically determined ecotypic differentiation, or, in an extreme case, there could be several cryptic species within *Sphagnum warnstorfii*. Considerable attention is today given to “the species problem,” and there is widespread application of phylogenetic approaches to the circumscription of species. Nevertheless, most species are distinguished because they look different. In small, structurally simple plants such as bryophytes, their morphology can be misleading because of a convergence with similar morphologies in unrelated plants (e.g., Holyoak and Pedersen 2007; Feldberg et al. 2010), coupled with high levels of morphological plasticity (which is common in the *Sphagnum* species, e.g., Karlin et al. 2008). Thus, a lack of morphological differentiation may not be indicative of underlying genetic patterns (e.g., Såstad 1999; Buryová and Shaw 2005). Morphologically cryptic lineages have been resolved within some widespread bryophytes, and these have been interpreted as cryptic species (reviewed in Shaw 2001; Fernandez et al. 2006; and Ramaiya et al. 2010). In aquatic or semiaquatic groups like *Sphagnum*, sorting out environmentally induced phenotypic modifications from genetically fixed traits, potentially useful for species delimitation, is especially difficult.

*Sphagnum* have dominant a haploid generation like other bryophytes. Diploid generation is a capsule placed on a haploid pseudopodium. *S. warnstorfii* is dioicous, which influences the population structure. The clonal asexual reproduction is common (Frey and Kürschner 2011), and the dioicous *S. warnstorfii* forms sporophytes less often than monoicous species, but more frequently than previously expected (Sundberg and Rydin 2002; Gunnarsson et al. 2007). The outbreeding of dioicous species results in greater genetic variability. Frequent sporophytes, together with high spore production and the ability for long-range dispersal (e.g., Sundberg 2013), could ensure the exchange of genetic information among populations at the regional scale, for example within Central Europe (Szövényi et al. 2012). The individual clones are supposed to be very long-lived and cover large areas (Cronberg 1996) or grow intermixed in small patches (Stenøien and Såstad 1999; Gunnarsson et al. 2007). *Sphagnum* in general differs from other bryophytes by the active dispersion of spores from the capsule (based on the mechanism of overpressure in the capsule; Ingold 1965). 19,240,000 spores are in one capsule (Sundberg and Rydin 2002), and the spores are relatively large compared to other perennial bryophytes (Hill et al. 2007). Spores are dispersed predominantly by the wind; the majority of spores drop up to 3.2 m from the capsule, but we can suggest traveling over longer distances with the help of thermal updrafts (Sundberg 2005). Nevertheless, there are certain dispersal limitations at the coarsest scales (Szövényi et al. 2008), at least for some species (Snäll et al. 2004). The founder effect, resulting from a dispersal at a large distance with the consequent inbreeding and high geographic isolation, could then lead to losses of heterozygosity in some isolated areas (Karlin et al. 2012, 2013).

The local population structure has been described in a few species of the *Sphagnum* subgenus *Acutifolia* (e.g., Shaw and Srodon 1995; Cronberg 1998; Natcheva and Cronberg 2003; Gunnarsson et al. 2007), the subgenus *Subsecunda* (e.g., Shaw et al. 2008; Ricca et al. 2011), the subgenus *Cuspidata* (Såstad et al. 1999, 2000; Stenøien and Såstad 1999; Szurdoki et al. 2014), and the subgenus *Sphagnum* (Thingsgaard 2001). Most studies were focused on calcifuge species, while calcium-tolerant species have been studied only marginally. Natcheva and Cronberg (2007) and Flatberg et al. (2006) found hybrids between calcium-tolerant S*phagnum warnstorfii* and various calcifuge species from the section *Acutifolia*. The population genetic structure was described in previous studies of *Sphagnum* using a broad range of molecular markers. DNA-based methods applied to bryophytes included nucleotide sequencing (e.g., Gunnarsson et al. 2007) and a variety of so-called fingerprinting methods (e.g., Zartman et al. 2006) including microsatellites. Microsatellites and nucleotide sequencing have been used most often in population studies of *Sphagnum* (e.g., Johnson et al. 2012; Szövényi et al. 2012; Karlin et al. 2014).

The aim of our study was to describe the genetic structure of the *Sphagnum warnstorfii* populations distributed along the pH/calcium gradient in the Hercynian, West Carpathian, and the Balkan mountain regions (central southeastern Europe). We collected two original and rigorously sampled datasets, including plant material and water chemistry, covering uniformly the pH/calcium gradient in the Hercynian mountains (Czech Republic) and in the Inner West Carpathians (Slovakia). In addition, we analyzed samples from refugial fens from Bulgaria (Hájková and Hájek 2007) accompanied by a directly measured pH and conductivity and a set of individual samples from other parts of the world.

The major questions this research addresses are as follows: (1) Are there differences in the levels of genetic variability between and within the regions that were studied in greatest detail (central southeastern Europe)? (2) Are plants from other regions (e.g., other parts of Europe, North America) genetically divergent from those from central southeastern Europe? (3) Is there a relationship between the genetic and physical distance separating the plants, that is, is there evidence of isolation by distance, reflecting dispersal limitations? (4) Is there a relationship between ecological distance, especially habitat pH/calcium levels, and genetic divergences that may reflect ecotypic differentiation?

## Materials and Methods

### Plant sampling

**Figure 2:**
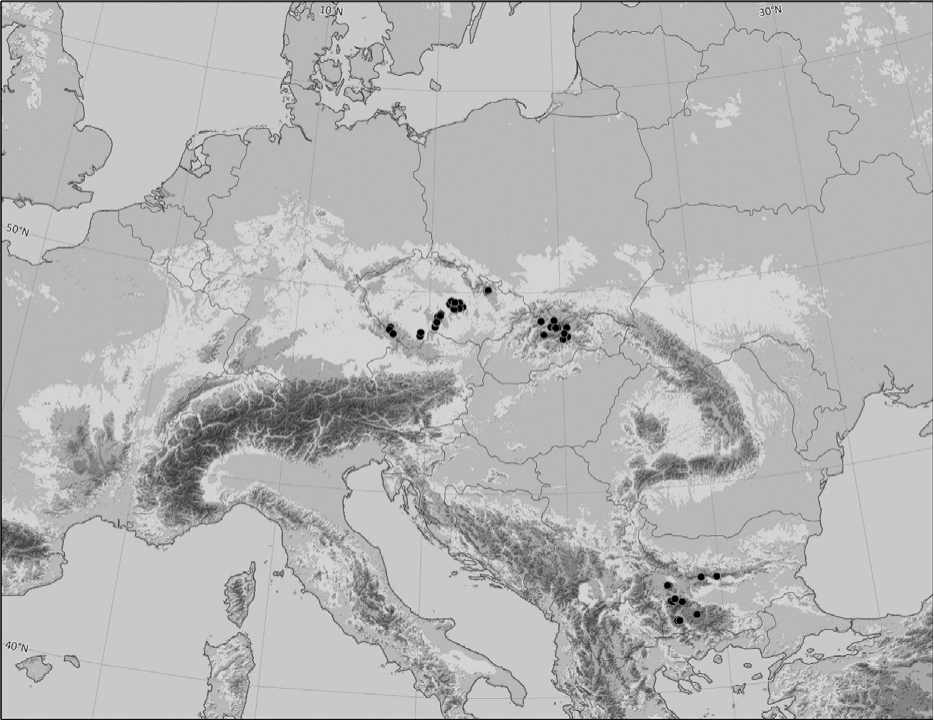
Map of sampled populations of *Sphagnum warnstorfii* in the Hercynian, West Carpathian, and Bulgarian mountains with detailed ecological data available (subset CZ-SK-BG).

We collected an original dataset in the Hercynian (Czech Republic) and West Carpathian mountains (Slovakia), using a formal sampling protocol and stratified selection of sites (Fig.2), equally representing the major Central European fen types delimited along the pH/calcium gradient (Hájek et al. 2006). The sampling area of one sampling site depended on the moss cushion size, but did not exceed 30 × 30 cm. The sampled moss plants were therefore collected within a distance of several cm (usually around 10 cm). For each sampling site (moss cushion), we determined the water pH, conductivity (using portable instruments, standardized to 20°C, and the conductivity caused by H^+^ ions was subtracted), and water chemistry (Ca^2+^, NH^4+^, NO^3−^, PO^4^, Na^+^, K^+^, Mg^2+^, Fe^III^; measured using atomic absorption spectrometry). Multiple samples from some populations (1–9 samples per moss cushion; 1–3 cushions per fen) were included to assess the genetic variations at more local scales. This dataset has the code CZ-SK throughout this paper.

For some analyses, we enlarged this dataset by more geographically distant samples. For the Balkans (Bulgaria), we utilized data for pH and conductivity from a previous ecological study (Hájková and Hájek 2007). The water pH and conductivity were measured the same way as in this study. The dataset enlarged by the Bulgarian samples has the code CZ-SK-BG. Finally, we used herbarium specimens to add data from other parts of the world in order to cover a large spatial scale.

All the samples included in the molecular analysis are kept in the Masaryk University herbarium (BRNM), in the Duke University Herbarium (DUKE), or private herbariums of authors. The locations for the vouchers are indicated in Appendix S1. A total of 335 samples of *S. warnstorfii* from 120 populations were included in the genetic analysis. Our oldest sample, from which microsatellites were amplified, was collected in 1977. The individual stems from which DNA was extracted were placed in small envelopes after a portion of the capitulum was sampled, and returned to the herbarium packet containing the rest of the collection. This allowed identifications and the morphological features to be checked after the genetic analyses were conducted, where necessary.

### Molecular methods

DNA extraction was accomplished according to the protocol of Doyle and Doyle (1987), modified as described at http://biology.duke.edu/bryology/protocols.html. The microsatellite genotyping protocols were described by Ricca et al. (2008). The primer sequences for microsatellite amplification were provided by Shaw et al. (2008). Initially, a sample of six individuals (two each from the Czech Republic, Slovakia, and Bulgaria) and a set of 18 microsatellite markers were used for screening the marker variability. The microsatellites were amplified in 8 *μ*L multiplexed reactions, each targeting a set of three loci, for a total of 12 loci (loci 1, 3, 9, 10, 14, 17, 18, 19, 20, 22, 29, and 30, numbered as in Shaw et al. 2008). The PCR products were diluted in sterile water, and 1.2 *μ*L of the dilution was mixed with GS500 size standard and Hi-DiTM Formamide (Applied Biosystems, Life Technologies Corporation, Carlsbad, CA) for electrophoresis on an ABI 3730 sequencer. Size determinations and genotype assignments were made using a GeneMarker, version 2.4.0 (Softgenetics, State College, PA).

### Data analysis

Fifteen samples with six or more missing loci were omitted. The final dataset included 320 samples from around the Northern Hemisphere (Appendix S1, Table2). For the analyses, we used two basic datasets; the dataset labeled CZ-SK included all the field-collected samples (210 samples) from the Czech Republic and Slovakia, and the dataset CZ-SK-BG included all those plus the samples from Bulgaria (269 samples). The microsatellite variation was analyzed using GenAlEx, version 6.5 (Peakall and Smouse 2006). Fragment sizes were coded as “codominant data,” and microsatellite repeat numbers were not calculated. The principal coordinates analyses (PCoAs) were computed for the full dataset of the unique genotypes (totally 135 samples). The PCoA was calculated from the genetic distance matrices using covariance matrices with data standardization, and missing data were not interpolated. We deleted samples with more than six missing loci (15 samples) and did not interpolate the genotypes in GenAlEx. Genetic distances were calculated using a dissimilarity matrix (Jaccard's Distance). This is the simplest model for microsatellite distances and does not involve the assumption that the loci evolve according to a substitution model (e.g., stepwise mutation). The analysis of molecular variance (AMOVA) was computed for dataset CZ-SK-BG (samples that did not have coordinates and samples that had only one sample per locality were removed; the dataset included 269 samples from 68 localities). The nested AMOVA was computed to examine the variations among the countries (the Czech Republic, Slovakia, Bulgaria), among the localities (separated fens = populations), and within localities. The percentages of total variance at each level and PhiPT, an analogue of the fixation index (FST), were estimated using GenAlEx. The significance of all the values was tested using 1000 random permutations. The percentage of clonality for the CZ-SK-BG dataset was computed using a custom R script (Matthew G. Johnson, DUKE University).

**Table 2:** Numbers of samples in particular datasets used in different analyses

Dataset	Clones included yes/no	“Outliers” included yes/no	*N*	Analysis
Full dataset	y	y	320	–
Full dataset	n	y	135	PcoA (Fig.4), Structure (Fig.3)
Full dataset	n	n	115	PcoA (Fig.5), Structure
CZ-SK-BG	y	y	269	AMOVA (Tab. 2), Spatial autocorrelations (Table3, Fig.6), Clonality
CZ-SK-BG	n	n	71	PCoA, Linear regression (Fig.8)
CZ-SK	y	y	210	–
CZ-SK	n	n	51	PCoA, Linear regression (Fig.7)

To test the correlations between genetic (Jaccard's) and geographic distances among individuals, Mantel tests based on Pearson's correlations were calculated using GENALEX. Spatial autocorrelations for assessing the relationships between the geographic and genetic distances between populations were also accomplished with GENALEX. The distance classes were created using the “even sample sizes” method, so that each class had an equal number of comparisons. The average values for each distance class were computed, and standard the errors for the multilocus estimates of the kinship coefficients per distance class were estimated using a jackknife procedure over the loci. The significance of all the coefficients was tested using 1000 random permutations within distance classes. Because we have exact coordinates only for samples from the Czech Republic, Slovakia, Bulgaria, and a few samples from North America, we divided the analysis of those samples into two parts. First, the autocorrelation analyses were run on the data from the dataset CZ-SK-BG; additional analyses were carried out with samples from North America only.

We tested for a genetic admixture among the samples and the correspondence of the genetic structure with defined regions using STRUCTURE, version 2.3.2 (Pritchard et al. 2000), in the final dataset (only unique genotypes were included; totally 135 samples). This program applies to a Bayesian model-based clustering method, which uses a Markov chain Monte Carlo (MCMC) algorithm to organize genetically similar individuals into clusters using multilocus genotype data. Admixture analyses using STRUCTURE were run with *K* = 2 through *K* = 9, with 10 replicate runs of 1 million generations (following a burn-in of 250,000 generations) at each value of *K*. The optimum number of clusters (*K*) was assessed using the Δ-*K* method (Evanno et al. 2005). Replicate runs within each value of *K* were summarized using CLUMPP 1.1.2 (Jakobsson and Rosenberg 2007) and visualized using a custom R script (Matthew G. Johnson, DUKE University).

We also tested for correlations between multilocus genotype and ecological variations in the datasets CZ-SK-BG (water pH and conductivity) and CZ-SK (water pH, conductivity, and 18 variables of water chemistry). The PCoA coordinates for the first three axes were calculated in GENALEX, using the same way as in the previous analyses, and transferred in R (version 2.15.3). For modeling and testing the relationship between ordination scores and environmental factors, we used the function *envfit* (package *vegan* 2.0-10; Oksanen et al. 2013), which performs a permutation test of the significance of the linear regression with 999 permutations. In the case of the CZ-SK dataset, where 20 intercorrelated environmental parameters were tested, the method of forward selection of the significant explanatory variables was used to avoid presenting falsely significant results in multiple tests. The significant linear regressions were visualized in ordination plots in R.

## Results

### Genetic structure

The final dataset included 11 loci (locus 3 did not work for *S. warnstorfii*). Locus 1 was almost monomorphic, loci 17, 18, 19, 20, 22, 29, and 30 had 5–9 alleles, and loci 9, 10, and 14 were highly variable with 15–23 alleles (see Appendix for details). 135 unique multilocus genotypes were identified in the final dataset of 320 gametophytes. The analysis performed on the CZ-SK-BG dataset showed 71 unique multilocus genotypes of 269 gametophytes (Table1), and 49 of them were distributed among localities. The same genotype occurred at widely separated sites, for example, different continents and regions. Of the 63 localities with more than one sample (dataset CZ-SK-BG), 16 had multiple genotypes, which formed the same cushion in most cases. Six localities had at least six samples; of these, three populations contained two genotypes and the other three only one genotype. One locality contained three very different genotypes in a single cushion.

AMOVAs (Table3) indicated a low genetic differentiation within *S. warnstorfii* among the Czech Republic, Slovakia, and Bulgaria (4% of the total variation). A high level of the genetic differentiation was at the locality level (among fens) (39%), and the majority of the intraspecific genetic variability occurred within fens (57%). All the components of variations were significant (*P *< 0.01). Several samples had distinct genetic profiles (see the Bayesian analysis below); however, the removal of these samples did not erase the significant genetic structure (results not shown).

**Table 3:** Analysis of molecular variance (AMOVA) in *Sphagnum warnstorfii* within the Czech Republic, Bulgaria, and Slovakia (CZ-SK-BG dataset; df = degrees of freedom, SS = sum of squares, MS = mean sums of squares)

Source	df	SS	MS	Estimated variance	Percentage of variation	Fixation index	*P*-value
(A) Three-level AMOVA for countries and individual fens							
Among countries	2	26.048	13.024	0.09	0.04	PhiRT = 0.038	<0.001
Among fens within countries	64	315.036	4.922	0.928	0.39	PhiPR = 0.407	<0.001
Within fens	193	261.327	1.354	1.354	0.57	PhiPT = 0.429	<0.001
(B) Among and within moss patches in one fen							
Among patches	66	379.663	5.752	1.868	0.91	–	–
Within patches	133	25.167	0.189	0.189	0.09	PhiPT = 0.908	<0.010

**Figure 3:**
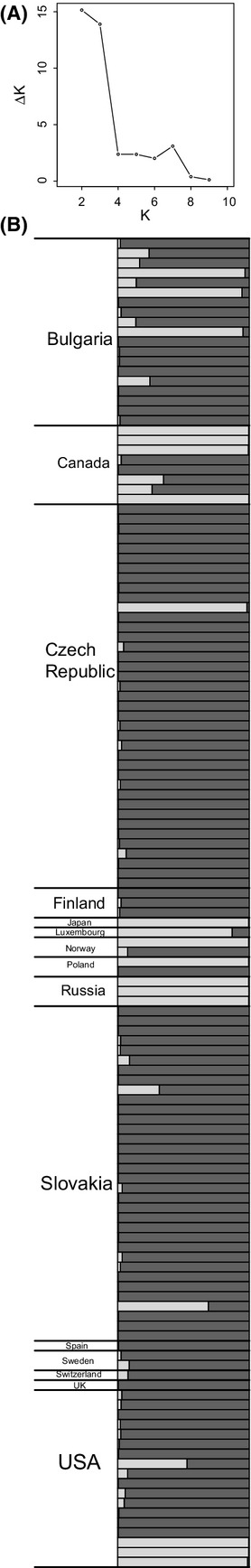
Plot of Δ-*K* in relation to number of clusters resolved in STRUCTURE analyses (A). Population structure of 135 plants of *Sphagnum warnstorfii* using the program STRUCTURE for the full dataset without clones. The graph shows the estimated membership for each individual in each cluster for *K* = 2 (the best K according to Δ-*K*). The individuals are grouped according to regions (B).

The Bayesian analysis of the complete dataset (with 135 unique genotypes) using STRUCTURE produced consistent results for *K* = 2 (Fig.3). Thus, the analysis identified two main genetic clusters within *S. warnstorfii*. 115 samples belonged to one cluster; only 20 belonged to the second cluster (“outlier” genotypes, “outlier” cluster). Sixty-five percent of all the samples seemed to be genetically pure (<1% of admixture) for one or the other genetic cluster. The majority of the samples showed <10% of genetic admixture in these two clusters, seven samples showed 10–30% of genetic admixture, and four samples showed a high level of admixture. The genetic structure (clusters) did not clearly correspond to geographic regions (continents, countries) or to measured environmental characteristics (Ca, pH, conductivity, fen type). The exact nature of the admixture in these individuals would require a more detailed analysis beyond the scope of this paper.

The STRUCTURE analysis without the 20 “outlier” genotypes resolved by the first analysis identified two main genetic clusters (*K* = 2), but all the individuals were admixtures between 30% and 70% for the first cluster. In this case, we should assume that the value of *K* is inappropriate and there is essentially no clustering in the dataset. This is very similar to the PCoA results and AMOVA results, which showed only 4% variability among the countries. We should conclude that the remaining samples make up only one wide population, with a very limited geographic structure.

**Figure 4:**
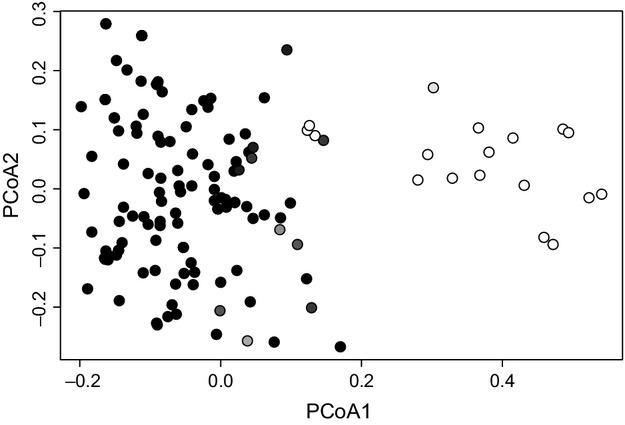
Principal coordinates analysis (PCoA) comparing 135 multilocus haploid genotypes of *Sphagnum warnstorfii* across 12 microsatellite markers for the full dataset without clones. The first two principal coordinates account for 46.9% and 15.6% of the total genetic variation. Plants are colored according to STRUCTURE results: first cluster – black, second cluster (“outliers”) – white, admixtures – gray.

**Figure 5:**
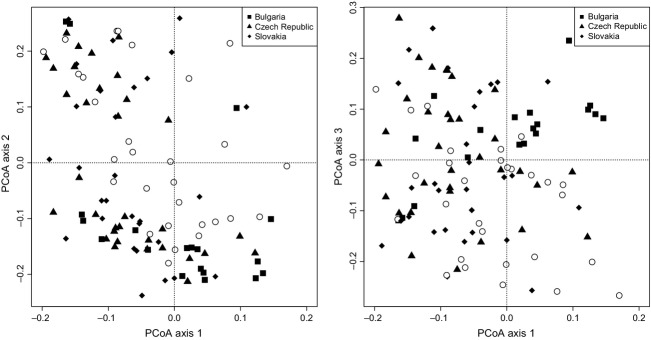
Principal coordinates analysis (PCoA) comparing 115 multilocus haploid genotypes of *Sphagnum warnstorfii* across 12 microsatellite markers for the full dataset without clones and “outliers.” The first three principal coordinates account for 26.49%, 20.16%, and 16.39% of the total genetic variation. Samples from the Central Europe are marked according to the country of origin, and samples from rest of the world are in white circles.

The PCoA of the entire dataset (Fig.4) suggested only a weak structure among the samples from different populations. The first axis explained 46.9% of the total variance, and the second and third explained only 15.6% and 11.4%, respectively. The samples from Central Europe were not regionally isolated in the context of global *S. warnstorfii* genetic diversity. The groups of samples on the right side of the PCoA plot (Fig.4) are formed by the same individuals as in the “outlier” cluster resolved in the STRUCTURE analysis. Even if we omitted samples from the “outlier” cluster from the PCoA, the geographic distances were not correlated with the genetic matrix (see Fig.5 for details).

### Geographic and genetic relationships

**Figure 6:**
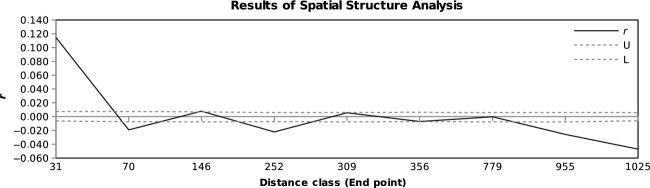
Result of spatial autocorrelation analysis of 269 individuals of *Sphagnum warnstorfii* from subset CZ-SK-BG based on microsatellites data. *r* – autocorrelation coefficient, U/L – upper and lower bounds of the 95% confidence interval for the null distribution (no isolation by distance).

Isolation by distance for whole dataset CZ-SK-BG was significant with *R*^2^ = 0.224 (*P *< 0.0001). When we split the data into blocks of geographic distances among individual samples, we should test the correlation between geographic and genetic distance within each block (Table4, Fig.6). There was a significant positive spatial autocorrelation at the local scale within an individual and very close fens (geographic distance class 0–31 km; *R*^2^ = 0.115, *P *< 0.001) due to the presence of clones within fens in the dataset CZ-SK-BG. These results corroborate the interpretation that cushions are largely clonal, as indicated by the AMOVA. At farther geographic distances (classes 31–70 km, 146–252 km, and especially 955–1025 km), the correlation was significantly negative. At those distances, there was no isolation by distance – the samples which were very far apart geographically could be very similar genetically (Fig.5). Values in other distance classes were insignificant, implying that the genetic similarity between the samples is independent of their geographic distance.

**Table 4:** Summary of spatial autocorrelation analysis of 269 individuals of *Sphagnum warnstorfii* from the CZ-SK-BG dataset based on microsatellite data. Kinship coefficients for dominant markers were calculated for each distance class (*n*). The first distance class refers to the correlation within populations. *n *= number of pairwise comparisons with a geographic distance less than or equal to upper bound of distance class, *r *= autocorrelation coefficient, U/L = upper and lower bounds of the 95% confidence interval for the null distribution (no isolation by distance), and the error bars were calculated by permuting the samples 1000 times. A significantly positive R would indicate isolation by distance within that distance class

*n*	3818	3326	3340	3356	3348	3360	3368	3338	3393
Distance Class (End Point; km)	31	70	146	252	309	356	779	955	1025
*r*	0.115	−0.019	0.008	−0.022	0.006	−0.007	0.000	−0.026	−0.047
U	0.007	0.007	0.007	0.006	0.006	0.006	0.006	0.006	0.006
L	−0.006	−0.007	−0.008	−0.007	−0.008	−0.007	−0.008	−0.008	−0.006
*P* (*r*-rand ≥ *r*-data)	**0.001**	1.000	0.011	1.000	0.044	0.973	0.543	1.000	1.000
*P* (*r*-rand ≤ *r*-data)	1.000	**0.001**	0.990	**0.001**	0.957	0.028	0.458	**0.001**	**0.001**

*P* values in bold indicate significant result.

The results from an autocorrelation analysis carried out with samples from North America correspond with results from Europe. There are similar levels of spatial autocorrelation when the divergent genotypes are removed (results not shown).

### Ecological and genetic relationships

**Figure 7:**
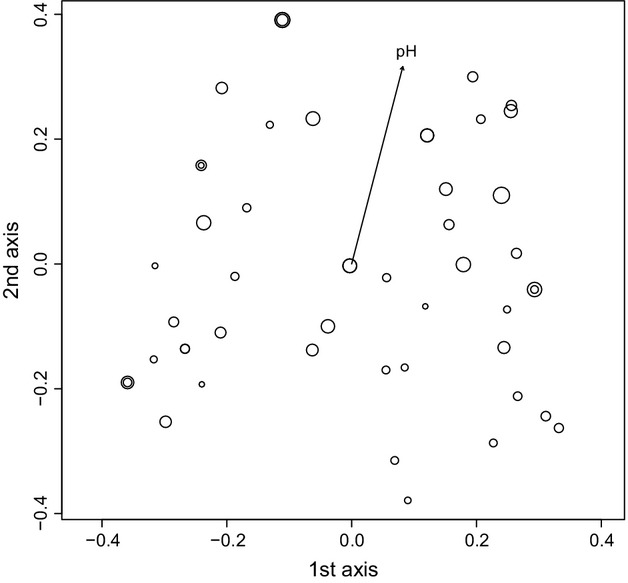
Principal coordinates analysis (PCoA) of 51 multilocus haploid genotypes of *Sphagnum warnstorfii* for the CZ-SK dataset with significant linear regression of pH (*R*^2^ = 0.1869, *P *< 0.05). The first three principal coordinates account for 14.5%, 11.8%, and 9.0% of the total genetic variation. The arrow indicates the direction of the significant linear regression, and size of the circles is proportional to the pH value.

**Figure 8:**
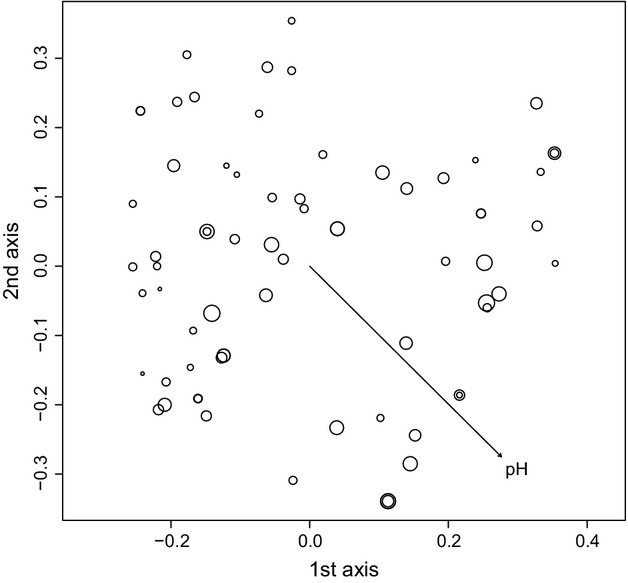
Principal coordinates analysis (PCoA) of 71 multilocus haploid genotypes of *Sphagnum warnstorfii* for the CZ-SK-BG dataset with significant linear regression of pH (*R*^2^ = 0.1002, *P *< 0.05). The first three principal coordinates account for 14.22%, 11.77%, and 9.07% of the total genetic variation. The arrow indicates the direction of the significant linear regression, and size of the circles is proportional to the pH value.

When we omitted samples from the “outlier” cluster from the PCoA, the first three axes of the genetic PCoA of the CZ-SK dataset (51 samples) correspond to 14.5%, 11.8%, and 9.0% of the total genetic variance, respectively. The only significant explanatory variable in the summary test of 20 environmental parameters was water pH (*R*^2^ = 0.1869, *P *< 0.05; Fig.7). Similar results were revealed in the CZ-SK-BG dataset (71 samples), whose genetic PCoA axes corresponded to 14.22%, 11.77%, and 9.07% of the total genetic variance. The only significant regression was again that for water pH (*R*^2^ = 0.1002, *P *< 0.05; Fig.8).

## Discussion

### The effect of pH

Our data suggest that despite the high dispersal ability of peatmosses (Sundberg 2013), confirmed also by a poor spatial signals in our datasets, the genetic structure of the *S. warnstorfii* population partially correlates with pH independently of geographic position. A high dispersal capacity may cause specifically adapted populations to occur over a wide geographic scale and may even meet, sympatrically, in one fen. The meeting of differently adapted populations could lead to a redistribution in the genetic variability and hence a formation of a cline of genetic variation along the pH gradient. The strong environmental filter of pH and calcium in rather small fen islands (Horsák et al. 2012) supports only a part of the genetic variability in each fen, maintaining an isolation by environment (Orsini et al. 2013), and hence a pH-related genetic structure independent of geography. The phenotypic traits that help the peatmoss gametophyte to survive under high pH/calcium levels involve unspecified physiological mechanisms (e.g., avoiding calcium input to the cytoplasm) or an ability to create hummocks elevating above the calcium-rich groundwater (Granath et al. 2010). The signs of local adaptation within bryophytes reported by Jules and Shaw (1994), Vanderpoorten and Tignon (2000), Szövényi et al. (2009), or Hutsemekers et al. (2010) were therefore confirmed by our study. On the other hand, a lack of geographic variation on larger scales does not support the predictions of the species pool hypothesis about each region harboring a specific ecotype of a species dependent on regional habitat availability (Hájková et al. 2008). Hence, our data rather support an idea that well-dispersing organisms such as spore-producing plants or protists (Hájek et al. 2011) may form single metapopulations and metacommunities operating on a large-scale rather than the idea about particular regional metacommunities suggested by Telford et al. (2006). We, however, demonstrated that if there is a strong environmental filter, this pattern probably does not exclude the existence of isolation by adaptation.

Rather than explaining differences between particular regions, different genotypes may explain the rather wide niche of *S. warnstorfii*. The occurrence of *S. warnstorfii* at different levels of base saturation in different regions could be thus explained rather by competitive release or the interference between individual ecological factors such as pH and calcium (Hájek et al. 2006, 2014), or between water level and calcium (Granath et al. 2010; Hájek et al. 2014). The interaction of these variables with iron (e.g., Aggenbach et al. 2013; Hájek et al. 2014) may be important as well.

### What is the major driver of genetic variation in *S. warnstorfii*?

The effect of pH discussed above, however, explained only a relatively small part of variability in our microsatellite data (Figs.5, 7, 8). The question remains as to what is the major driver of the observed genetic variance in our data? The first possible explanation is spatial autocorrelation in genotypes at small, but not at larger distances. However, even samples from the same fens had been diversified along both the first and the second PCoA axis.

The second possible explanation is the cline of genetic variation caused by introgression due to repeated back-hybridizations. Already the first run of STRUCTURE analysis revealed a small number of populations that were significantly different from the others throughout the Northern Hemisphere. A detailed study of the morphology of these genetically divergent samples indeed showed some transitional characteristics to the other species of the subgenus *Acutifolia*, especially *S. subnitens, S. rubellum*, and *S. capillifolium*. The plants had small ringed pores on branch leaves, but unusually acute stem leaves. These morphological features, together with this genetic difference could suggest a possible hybrid origin(s) of these samples (E. Mikulášková in prep.). These samples were removed from further analyses, but back-hybridization may take place forming a cline of genetic variations not corresponding well with morphology. Interspecific hybridization has been already documented in the subgenus *Acutifolia* by Cronberg (1998), Flatberg et al. (2006), and Natcheva and Cronberg (2007). Microsatellite data further revealed hybridization within the subgenus *Cuspidata* (Szurdoki et al. 2014), and hybridization among species from different subgenera has even been shown (Karlin et al. 2009, 2014). The ability to hybridize may not be uniform across the subgenus *Acutifolia*, and the genetic data suggest that *S. rubellum* and *S. warnstorfii* tend to hybridize easily (Shaw and Cox 2005). The results of Natcheva and Cronberg (2003) from the Bulgarian high mountains even suggest a complete introgression of *S.** **rubellum* into other species of the subgenus *Acutifolia*. Hybridization might therefore explain why *S.** **warnstorfii* in Bulgarian high mountains occupy sites with lower calcium levels as compared to Central Europe (Hájková and Hájek 2007).

The third possible cause of unexplained genetic variance is the legacy of glacial refugia. During pleniglacials, a dry climate and frequent disturbances supported the existence of shallow brown-moss fens rather than bogs or *Sphagnum* hummocks (Gajewski et al. 2001). Indeed, *Sphagnum warnstorfii* are absent in the regions that experience extremely dry summers such as the lower altitudes of the Balkans (Daniels and Eddy 1985; Hájková and Hájek 2007) or the Central Asian mountains, which are considered as an analogy of the European glacials (Horsák et al. 2010). The distribution of *S.** **warnstorfii* during pleniglacials could be very fragmented, yielding genetic diversification because of isolation by distance, similar to the results of Natcheva and Cronberg (2003). More recently, a higher habitat connectivity and higher dispersal ability of the species would lead to dissolving this ancient diversification.

These hypotheses can be neither confirmed nor rejected without further investigation. The indirect evidence available at this moment supports, rather, the hypothesis of hybridization events.

### Negligible effects of geography

In our study, the genetic differentiation in *S. warnstorfii* was low (but nonzero) among the samples from different continents. Low levels or even a lack of genetic differentiation between plants on different continents occur in other bryophytes, including the *Sphagnum* species (see, e.g., Stenøien and Såstad 1999; Thingsgaard 2001; Stenøien et al. 2011). Some studies have shown a significant, yet weak, genetic differentiation between the intercontinental populations of peatmoss species (Stenøien and Såstad 1999; Szövényi et al. 2008; Karlin et al. 2013). Our results suggest that the North Atlantic Ocean is not an insurmountable barrier for gene flow in a *S. warnstorfii* metapopulation. Only a relatively small portion of the total genetic variability we documented in *S. warnstorfii* was explained by locations in different countries in Central Europe. Genetic variability was concentrated at the population (fen) level, mainly among separated cushions within fens. Closely similar genotypes were shared both among countries within Europe and between Europe and North America.

The similar genotypes are found both in the most densely sampled regions (Czech Republic, Slovakia, and Bulgaria) and all around the Northern Hemisphere. Contrary to this, Natcheva and Cronberg (2003) found a low number of widespread genotypes in the closely related species, *Sphagnum capillifolium*, in the high Bulgarian mountains. The unique genotypes have been distributed across all the regions as well. We explain these observed patterns by the high levels of gene flow across the Northern Hemisphere.

The spatial autocorrelation analyses show no general trends in correlation between genetic and geographic distances. A significant correlation was found only at small spatial scales, that is, for individual gametophytes within populations, or between geographically proximate populations. Significantly negative autocorrelations at large geographic scales support our conclusion that *S. warnstorfii* readily spreads over long distances, in agreement with the results from some other bryophytes that have been studied (see, e.g., Ramaiya et al. 2010). Individual colonies (cushions) within a site are largely of clonal origin, as they are mostly formed by a single multilocus genotype. This result is consistent with that of Shaw and Srodon (1995), Cronberg (1998), and Gunnarsson et al. (2007) on other species of *Sphagnum*. Individual populations may establish from multiple sources distributed regionally. As a consequence, genetic diversity within a fen is a subset of the genetic diversity within the region.

## Conclusions

1. As evidenced by the genetic structure of *S. warnstorfii,* the establishment of individual populations appears to arise from multiple sources. The genetic diversity within a fen is a subset of the genetic diversity within the region. Individual cushions within a fen are mainly of clonal origin.
2. There is no correlation between genetic and geographic distances among samples at a coarse spatial scale.
3. Although water pH explains a significant part of the genetic variation within *S. warnstorfii*, there is much more variation, which is difficult to explain. Its major part is possibly accounted for by a cline of genetic variation shaped by hybridization and back-hybridization with other species, especially *S. subnitens, S. rubellum*, and *S. capillifolium*. This unexplained variation can, however, also mirror a fine-scale spatial autocorrelation or a legacy of past genetic differentiation.
4. Our data support an idea that well-dispersing organisms such as spore-producing plants form one metapopulation operating at a large-scale, rather than the existence of particular regional metapopulations. This pattern, however, does not exclude the existence of local adaptations, because pH as a strong environmental determinant of peatmoss occurrence could filter particular genotypes from a species pool and hence could support isolation by environment.
5. Our results generally confirm the high dispersal ability of peatmosses, but simultaneously suggest that their ability to cope with a high pH/calcium level is at least partially determined genetically. Genetics may explain a rather wide niche of *S. warnstorfii*, but cannot explain differences in the pH niche between particular regions, as we initially expected.

## Data Accessibility

1. Full dataset of microsatellite data (Sample ID, sampling location, genotype at particular loci) uploaded as online Appendix.
2. Water chemistry data uploaded as online Appendix.
3. K3 STRUCTURE plot attached as online Supplementary data.

## Supporting Information

Appendix S1. Localities and ecological data + sample IDs and microsatellite data.
