# Supplementary material for: Local adaptations in bryophytes revisited: the genetic structure of the calcium-tolerant peatmoss Sphagnum warnstorfii along geographic and pH gradients
Source: Ecol Evol. 2014 Dec 19;5(1):229–42. doi: 10.1002/ece3.1351 (PMC4298450; doi:10.1002/ece3.1351)
Supplement: Supplementary file 1 [file ece30005-0229-sd1.pdf]

Appendix tables: localities and ecological data + sample IDs and microsatellite data

State	Locality name	nr. of genetic samples	WGS-84 N	WGS-84 E (W)	herbarium	Water chemism: pH	Conduct. 20°C	$N\text{-}NH_4^+$ µg/L	$N\text{-}NO_3^-$ µg/L	P-PO4 µg/L	SO_4^{2-} mg/L	Na^+ mg/L	Ca^{2+} mg/L	Ca+Mg mg/L	K^+ mg/L	Mg^{2+} mg/L	Fe^{III} mg/L
Bulgaria	Central Rhodopes – Near the jazovir Beglika	5	41.8247222	24.1230556	M. Hájek, BRNU	6.0	55.000	n/a	n/a	n/a	n/a	n/a	n/a	n/a	n/a	n/a	n/a
Bulgaria	Pirin – Izvorite	2	41.7147222	23.5430556	M. Hájek, BRNU	5.4	14.000	n/a	n/a	n/a	n/a	n/a	n/a	n/a	n/a	n/a	n/a
Bulgaria	Pirin – NW from Bezbog, Disilishko reka	5	41.7380556	23.5063889	M. Hájek, BRNU	6.0	28.000	n/a	n/a	n/a	n/a	n/a	n/a	n/a	n/a	n/a	n/a
Bulgaria	Pirin, Golyamo Valyavishko ozero	5	41.7147222	23.4697222	M. Hájek, BRNU	5.8	32.000	n/a	n/a	n/a	n/a	n/a	n/a	n/a	n/a	n/a	n/a
Bulgaria	Rila – Govedartsi	3	42.2522222	23.4466667	M. Hájek, BRNU	5.7	81.000	n/a	n/a	n/a	n/a	n/a	n/a	n/a	n/a	n/a	n/a
Bulgaria	Rila – Kazanishka reka (Kostenecki Balkan)	2	42.1677778	23.6769444	M. Hájek, BRNU	5.7	34.000	n/a	n/a	n/a	n/a	n/a	n/a	n/a	n/a	n/a	n/a
Bulgaria	Rila – Malyovitsa	5	42.1830556	23.3711111	M. Hájek, BRNU	5.5	14.000	n/a	n/a	n/a	n/a	n/a	n/a	n/a	n/a	n/a	n/a
Bulgaria	Rila – Sedemse Ozera	4	42.2036111	23.3125000	M. Hájek, BRNU	4.3	26.000	n/a	n/a	n/a	n/a	n/a	n/a	n/a	n/a	n/a	n/a
Bulgaria	Stara Planina – Botev, Kafadikildi	5	42.7125000	24.8930556	M. Hájek, BRNU	4.3	6.000	n/a	n/a	n/a	n/a	n/a	n/a	n/a	n/a	n/a	n/a
Bulgaria	Stara Planina – Vartopa (pod Veženem)	5	42.7305556	24.3705556	M. Hájek, BRNU	5.2	39.000	n/a	n/a	n/a	n/a	n/a	n/a	n/a	n/a	n/a	n/a
Bulgaria	Vitosha - between Aleko and Platoto	3	42.5880556	23.2872222	M. Hájek, BRNU	6.1	80.000	n/a	n/a	n/a	n/a	n/a	n/a	n/a	n/a	n/a	n/a
Bulgaria	Vitosha – 3km S of Zlatni Mostove	2	42.6016667	23.2391667	M. Hájek, BRNU	5.5	89.000	n/a	n/a	n/a	n/a	n/a	n/a	n/a	n/a	n/a	n/a
Bulgaria	Vitosha – above Zvjezditsa challet	3	42.5813889	23.2297222	M. Hájek, BRNU	5.0	28.000	n/a	n/a	n/a	n/a	n/a	n/a	n/a	n/a	n/a	n/a
Bulgaria	Vitosha – Kapaklivec	3	42.5913889	23.2863889	M. Hájek, BRNU	5.4	65.000	n/a	n/a	n/a	n/a	n/a	n/a	n/a	n/a	n/a	n/a
Bulgaria	Vitoska – between Sredetsa and Lavcheto	2	42.5844444	23.2563889	M. Hájek, BRNU	n/a	n/a	n/a	n/a	n/a	n/a	n/a	n/a	n/a	n/a	n/a	n/a
Bulgaria	Vitoska – Cherni Vrah	5	42.5491667	23.2794444	M. Hájek, BRNU	n/a	n/a	n/a	n/a	n/a	n/a	n/a	n/a	n/a	n/a	n/a	n/a
Czech Republic	Borová: Pod Koupalištěm	3	49.7396036	16.1482717	E. Mikulášková	5.8	173.933	445.139	27.699	303.541	n/a	20.100	16.670	20.281	18.085	3.611	28.510
Czech Republic	Borova: Pod nadrazim	3	49.7427461	16.1650569	E. Mikulášková	6.2	443.813	n/a	n/a	n/a	n/a	n/a	n/a	n/a	n/a	n/a	n/a
Czech Republic	Buchtovka	3	49.7737478	15.8123058	E. Mikulášková	6.2	216.667	664.199	442.032	123.926	n/a	10.465	12.580	16.231	11.720	3.651	50.920
Czech Republic	Častá	8	48.9202900	13.6707700	E. Mikulášková	n/a	n/a	n/a	n/a	n/a	n/a	n/a	n/a	n/a	n/a	n/a	n/a
Czech Republic	Čermákovy louky	3	49.3852222	15.3295833	E. Mikulášková	6.0	108.133	234.504	276.576	80.298	n/a	7.295	5.158	7.249	19.150	2.091	71.040
Czech Republic	Damašek	3	49.7183317	16.1271269	E. Mikulášková	5.7	93.367	141.060	553.399	100.167	n/a	5.055	14.240	16.631	5.280	2.391	1.511
Czech Republic	Filipova Hut	3	49.0262475	13.5177069	E. Mikulášková	n/a	n/a	n/a	n/a	n/a	n/a	n/a	n/a	n/a	n/a	n/a	n/a
Czech Republic	Chvojnov	7	49.4072780	15.4191940	E. Mikulášková	5.7	118.600	188.512		91.728	n/a	4.622	17.090	19.808	3.409	2.718	34.480
Czech Republic	Kaliště 2A	3	49.2518611	15.2987222	E. Mikulášková	5.1	92.611	24.759	27.891	90.855	n/a	4.448	6.232	7.330	3.543	1.098	61.020
Czech Republic	Kaliště 2B	3	49.2501944	15.2968333	E. Mikulášková	5.7	87.167	58.154	96.070	102.925	n/a	3.554	4.560	6.247	9.965	1.687	32.270
Czech Republic	Kvilda	3	49.0821100	13.5545900	E. Mikulášková	n/a	n/a	n/a	n/a	n/a	n/a	n/a	n/a	n/a	n/a	n/a	n/a
Czech Republic	Louky u Černého lesa	3	49.5855000	15.9423333	E. Mikulášková	6.0	111.650	21.131	5.404	122.211	n/a	8.265	19.700	21.784	7.955	2.084	5.392
Czech Republic	Louky u Jeníkova	3	49.7536869	15.9607950	E. Mikulášková	6.6	167.367	117.406	533.350	161.484	n/a	13.155	14.650	21.115	5.475	6.465	4.017
Czech Republic	Malá Kotlina9	6	50.0410556	17.2103056	E. Mikulášková	7.2	45.300	n/a	n/a	n/a	n/a	n/a	n/a	n/a	n/a	n/a	n/a
Czech Republic	Mechové údolí 2	3	50.0090500	12.5145600	E. Mikulášková	6.7	78.000	n/a	n/a	n/a	n/a	n/a	n/a	n/a	n/a	n/a	n/a
Czech Republic	Mokřady pod Vlčkem	3	50.0400800	12.7360500	E. Mikulášková	7.2	214.000	29.851	12.348	33.732	0.560	3.251	3.655	38.186	0.143	34.531	2.187
Czech Republic	Na Oklice	3	49.4031389	15.3953056	E. Mikulášková	5.8	141.900	158.925	302.607	151.827	n/a	9.725	9.749	12.698	6.620	2.949	62.000
Czech Republic	Odranec 2	3	49.6114722	16.1397500	E. Mikulášková	6.1	111.867	25.930	58.606	46.309	n/a	4.096	4.075	5.287	19.310	1.212	14.540
Czech Republic	Plíčky	5	49.5658890	15.9743330	E. Mikulášková	6.5	248.000	40.770	790.260	125.789	n/a	4.857	30.520	34.915	1.630	4.395	3.084
Czech Republic	Ratajské rybníky	5	49.7700660	15.9377880	E. Mikulášková	6.3	152.000	121.177	79.357	170.871	n/a	11.555	41.860	49.561	3.618	7.701	181.700
Czech Republic	Řeka2	5	49.6661390	15.8529720	E. Mikulášková	6.6	327.333	27.688	173.193	139.347	n/a	4.723	55.240	56.787	3.075	1.547	9.135
Czech Republic	Suchdolské rašeliniště	3	49.1318611	15.2373333	E. Mikulášková	5.3	104.925	65.770	73.081	57.619	n/a	0.386	19.510	21.769	0.158	2.259	10.700
Czech Republic	Suchopýrek	3	49.6472508	16.2683350	E. Mikulášková	6.5	167.667	141.746	834.173	181.103	n/a	9.925	22.220	25.913	12.560	3.693	11.220
Czech Republic	Šimanovské rašeliniště	3	49.4501667	15.4470278	E. Mikulášková	5.7	109.800	108.540	293.548	168.756	n/a	12.770	3.440	5.442	3.682	2.002	6.708
Czech Republic	Švábov	7	49.3163330	15.3486390	E. Mikulášková	6.2	210.333	53.174	449.013	148.873	n/a	11.500	25.220	29.917	2.765	4.697	3.820
Czech Republic	Trhová Kamenice2	3	49.7825000	15.8385831	E. Mikulášková	6.2	239.770	n/a	n/a	n/a	n/a	n/a	n/a	n/a	n/a	n/a	n/a
Czech Republic	V Lísovech	3	49.2476667	15.2790000	E. Mikulášková	6.2	117.000	40.577	24.893	126.552	n/a	5.220	12.350	14.284	2.795	1.934	35.320
Czech Republic	V Rájích	3	48.9860000	14.7089167	E. Mikulášková	6.3	345.667	65.477		81.120	n/a	4.024	35.970	46.120	8.294	10.150	1.573
Czech Republic	Velká kotlina1	4	50.0593056	17.2421389	E. Mikulášková	6.6	25.700	n/a	n/a	n/a	n/a	n/a	n/a	n/a	n/a	n/a	n/a

Czech Republic	Velká kotlina2	3	50.0586667	17.2389167	E. Mikulášková	6.9	30.900	n/a	n/a	n/a	n/a	n/a	n/a	n/a	n/a	n/a	n/a
Czech Republic	Velká kotlina3	6	50.0580278	17.2380000	E. Mikulášková	7.1	66.300	n/a	n/a	n/a	n/a	n/a	n/a	n/a	n/a	n/a	n/a
Czech Republic	Vortová: Návesník	3	49.7114442	15.9277500	E. Mikulášková	5.8	87.585	n/a	n/a	n/a	n/a	n/a	n/a	n/a	n/a	n/a	n/a
Czech Republic	Zlámanec	3	49.7047061	15.9316469	E. Mikulášková	5.8	88.433	249.733	634.734	158.067	n/a	8.180	21.070	24.840	4.433	3.770	4.087
Czech Republic	Zlatá louka	3	49.7142925	15.7730281	E. Mikulášková	6.9	273.000	88.952	554.076	63.772	n/a	0.615	68.540	69.832	3.340	1.292	1.900
Czech Republic	Žemlička	3	48.8918333	14.6879167	E. Mikulášková	6.4	144.000	157.461	398.536	152.509	n/a	1.573	11.900	15.419	3.472	3.519	4.435
Slovakia	Gerlachov	3	49.1094167	20.2122500	E. Mikulášková	5.5	56.714	360.731	31.424	24.136	19.890	8.091	10.090	12.702	1.876	2.612	3.001
Slovakia	Havraník (Zlatno)	6	48.8185278	20.0629722	E. Mikulášková	6.8	460.413	0.001	18.850	0.001	110.277	8.850	50.087	67.334	1.080	17.247	1.101
Slovakia	Hnilecká jelšina (Dobš. ľad. jaskyňa)	7	48.8752222	20.2958611	E. Mikulášková	6.4	211.488	0.001	15.194	0.001	14.155	1.733	60.853	67.634	0.517	6.781	1.022
Slovakia	Hnilecká jelšina (Pusté pole)	1	48.8794440	20.2304170	E. Mikulášková	7.0	328.478	88.192	18.393	0.001	15.657	2.917	65.691	85.149	0.359	19.458	1.022
Slovakia	Jalovec	4	49.1329440	19.6282500	E. Mikulášková	6.1	63.401	79.749	12.757	0.001	12.987	2.846	14.241	16.213	0.916	1.972	0.628
Slovakia	Jochy	5	49.1208060	19.7731110	E. Mikulášková	5.4	50.596	17.076	23.556	12.362	21.680	3.201	4.250	6.015	0.644	1.765	2.720
Slovakia	Liptovská Lužná 1	6	48.9401111	19.3523611	E. Mikulášková	6.9	379.199	17.078	15.459	0.001	71.746	1.855	31.773	49.525	0.780	17.752	0.308
Slovakia	Liptovská Lužná 2	6	48.9312222	19.3469444	E. Mikulášková	6.0	71.855	6.536	16.431	0.001	2.882	1.103	5.289	10.337	0.305	5.048	0.324
Slovakia	Liptovská Lužná 3	3	48.9312222	19.3469444	E. Mikulášková	5.9	70.000	19.105	17.240	101.540	10.822	5.544	5.171	9.850	1.034	4.679	0.324
Slovakia	Liptovská Teplička 1	9	48.9639722	20.1065556	E. Mikulášková	6.8	181.750	0.001	0.001	0.001	12.853	2.065	34.610	47.636	0.144	13.026	0.146
Slovakia	Liptovská Teplička 2	3	48.9520556	20.1034722	E. Mikulášková	7.1	354.745	n/a	n/a	n/a	n/a	n/a	n/a	n/a	n/a	n/a	n/a
Slovakia	Podbanské	3	49.1110833	19.8804167	E. Mikulášková	5.0	49.657	41.655	266.774	12.362	7.770	5.323	7.283	9.162	1.107	1.879	3.001
Slovakia	Pribylina	3	49.1242222	19.8499444	E. Mikulášková	5.6	67.024	61.675	18.050	96.210	5.744	11.794	4.481	6.965	1.514	2.484	0.259
Slovakia	Pribylina - za Esperantem	9	49.1091111	19.8108333	E. Mikulášková	6.7	91.479	0.001	0.001	0.001	6.114	2.990	14.807	18.474	0.343	3.667	0.259
Slovakia	Puchmajerovej jazierko	5	49.2785000	19.2527500	E. Mikulášková	5.7	83.667	134.966	34.921	31.062	14.180	6.342	18.050	19.659	0.465	1.609	1.571
Slovakia	Švihrová 2	3	49.1110278	19.7711667	E. Mikulášková	5.1	64.524	96.731	23.556	29.330	9.170	5.265	5.878	4.350	0.963	1.801	2.720
Slovakia	Telgárt, Pšolinec 1	3	48.8535833	20.1815278	E. Mikulášková	6.1	510.832	29.894	0.001	0.001	9.166	9.758	113.897	143.956	2.903	30.059	0.768
Canada/Alberta	Rt. 813 N of Athabasca	1	54.0963333	-113.2585000	Duke, Nr. 96613	n/a	n/a	n/a	n/a	n/a	n/a	n/a	n/a	n/a	n/a	n/a	n/a
Canada/British columbia	Canada-Alaska border	1	59.6373850	-135.1524930	Duke, Nr. 11206x	n/a	n/a	n/a	n/a	n/a	n/a	n/a	n/a	n/a	n/a	n/a	n/a
Canada/British columbia	Canada-Alaska border	1	59.6541667	-135.1266667	Duke, Nr. 126531	n/a	n/a	n/a	n/a	n/a	n/a	n/a	n/a	n/a	n/a	n/a	n/a
Canada/British columbia	Vancouver Island	1	50.1666679	-127.7500000	Duke, Nr. 96645	n/a	n/a	n/a	n/a	n/a	n/a	n/a	n/a	n/a	n/a	n/a	n/a
Canada/New Brunswick	Kouchibouguac National Park	1	46.8833351	-64.9499969	Duke, Nr. 96725	n/a	n/a	n/a	n/a	n/a	n/a	n/a	n/a	n/a	n/a	n/a	n/a
Canada/New Foundland	On Hwy. 432	1	51.0483330	-56.7966670	Duke, Nr. 175713	n/a	n/a	n/a	n/a	n/a	n/a	n/a	n/a	n/a	n/a	n/a	n/a
Canada/Quebec	Isles de Mingan	1	50.2166672	-63.6500015	Duke, Nr. 96775	n/a	n/a	n/a	n/a	n/a	n/a	n/a	n/a	n/a	n/a	n/a	n/a
Canada/Yukon	Larsen Creek	1	60.2000008	-125.5333328	Duke, Nr. 96838	n/a	n/a	n/a	n/a	n/a	n/a	n/a	n/a	n/a	n/a	n/a	n/a
Finland	Suurisuo Mire	1	60.9833336	24.7999992	Duke, Nr. 18447	n/a	n/a	n/a	n/a	n/a	n/a	n/a	n/a	n/a	n/a	n/a	n/a
Finland	Oulanka Nat. Park	1	66.3499985	29.3166676	Duke, Nr. 65152	n/a	n/a	n/a	n/a	n/a	n/a	n/a	n/a	n/a	n/a	n/a	n/a
Finland	Heinisuo Fen	1	61.0333330	25.0383330	Duke, Nr. 18443	n/a	n/a	n/a	n/a	n/a	n/a	n/a	n/a	n/a	n/a	n/a	n/a
Japan	Nemuro vicinity. Ochi-ishi Wetland.	1	43.2241000	145.5075000	Duke, Nr. 157093	n/a	n/a	n/a	n/a	n/a	n/a	n/a	n/a	n/a	n/a	n/a	n/a
Luxembourg	Luxembourg	1	49.8579000	-6.5472800	Duke, Nr. 43	n/a	n/a	n/a	n/a	n/a	n/a	n/a	n/a	n/a	n/a	n/a	n/a
Norway	Stjordal, Hestoasen	1	63.4944000	11.0305000	Duke, Nr. 174728	n/a	n/a	n/a	n/a	n/a	n/a	n/a	n/a	n/a	n/a	n/a	n/a
Norway	Trondelag, Levanger Municipality: Upper Forra area	1	63.5812520	11.6568310	Duke, Nr. 18245	n/a	n/a	n/a	n/a	n/a	n/a	n/a	n/a	n/a	n/a	n/a	n/a
Poland	Rospuda basin, Szczebecka	3	53.9418410	22.8850080	E. Mikulášková	6.9	304.000	n/a	n/a	n/a	n/a	5.420	61.750	n/a	3.989	2.867	2.534
Poland	Plaine de Walcz	1	53.3121000	16.5187000	Duke, Nr. 174691	n/a	n/a	n/a	n/a	n/a	n/a	n/a	n/a	n/a	n/a	n/a	n/a
Russia	Ayon Island	1	69.2483000	154.6842000	Duke, Nr. AYOwarn12	n/a	n/a	n/a	n/a	n/a	n/a	n/a	n/a	n/a	n/a	n/a	n/a
Russia	Wrangel Island	2	71.2019000	179.5550000	Duke, Nr. WRAwarm3, WRAwarm10	n/a	n/a	n/a	n/a	n/a	n/a	n/a	n/a	n/a	n/a	n/a	n/a
Spain	Alto Aneu, Valle de Aran	1	42.7448500	0.7924200	Duke, Nr. 174693	n/a	n/a	n/a	n/a	n/a	n/a	n/a	n/a	n/a	n/a	n/a	n/a
Sweden	mountain Visjövalen	1	63.2833328	12.1833334	Duke, Nr. 18246	n/a	n/a	n/a	n/a	n/a	n/a	n/a	n/a	n/a	n/a	n/a	n/a
Sweden	Jamtlad, 2 km W of Mattmas	1	63.2833330	13.7333330	Duke, Nr. 18247	n/a	n/a	n/a	n/a	n/a	n/a	n/a	n/a	n/a	n/a	n/a	n/a
Switzerland	Scalettahorn	1	46.7000000	9.9166667	Duke, Nr. 139693	n/a	n/a	n/a	n/a	n/a	n/a	n/a	n/a	n/a	n/a	n/a	n/a
UK	Berwickshire	1	55.7934000	-2.3698000	Duke, Nr. 26694	n/a	n/a	n/a	n/a	n/a	n/a	n/a	n/a	n/a	n/a	n/a	n/a
USA/Alaska	Izembek National Wildlife Refuge	2	55.2666670	-162.8833330	Duke, Nr. 96425	n/a	n/a	n/a	n/a	n/a	n/a	n/a	n/a	n/a	n/a	n/a	n/a
USA/Connecticut	Holleran Swamp	1	42.0361111	-73.2000000	Duke, Nr. 138800	n/a	n/a	n/a	n/a	n/a	n/a	n/a	n/a	n/a	n/a	n/a	n/a

USA/Idaho	Secesh Summit	1	45.1833344	-115.9666672	Duke, Nr. 3048	n/a	n/a	n/a	n/a	n/a	n/a	n/a	n/a	n/a	n/a	n/a	n/a
USA/Maine	Penobscot Co.	1	46.1333333	-68.6833333	Duke, Nr. 213529	n/a	n/a	n/a	n/a	n/a	n/a	n/a	n/a	n/a	n/a	n/a	n/a
USA/Maine	WELS Townships	1	46.1277778	-68.6916667	Duke, Nr. 96445	n/a	n/a	n/a	n/a	n/a	n/a	n/a	n/a	n/a	n/a	n/a	n/a
USA/Michigan	Summberbee Swamp	1	45.9666667	-84.8000000	Duke, Nr. 96468	n/a	n/a	n/a	n/a	n/a	n/a	n/a	n/a	n/a	n/a	n/a	n/a
USA/Minnessota	USA/Minnessota	1	47.3166656	-91.1333313	Duke, Nr. 96470	n/a	n/a	n/a	n/a	n/a	n/a	n/a	n/a	n/a	n/a	n/a	n/a
USA/New Hampshire	Hulbert Swamp Preserve	1	44.9879000	-71.4418000	Duke, Nr. 68727	n/a	n/a	n/a	n/a	n/a	n/a	n/a	n/a	n/a	n/a	n/a	n/a
USA/New Jersey	Johnsonburg	1	40.9596300	-74.8987590	Duke, Nr. 96514	n/a	n/a	n/a	n/a	n/a	n/a	n/a	n/a	n/a	n/a	n/a	n/a
USA/Oregon	Blue Mountains, Olive Lake	1	44.7833328	-118.5833359	Duke, Nr. 11699	n/a	n/a	n/a	n/a	n/a	n/a	n/a	n/a	n/a	n/a	n/a	n/a
USA/South Carolina	Weston Lake	1	34.0380000	-80.8290000	Duke, Nr. 17459	n/a	n/a	n/a	n/a	n/a	n/a	n/a	n/a	n/a	n/a	n/a	n/a
USA/Utah	Unita Mountains, Wasatch National Forest	1	40.7008333	-110.8866667	Duke, Nr. 138888	n/a	n/a	n/a	n/a	n/a	n/a	n/a	n/a	n/a	n/a	n/a	n/a
USA/Vermont	White Cedar Swamp	1	44.5666667	-71.8333333	Duke, Nr. 67798	n/a	n/a	n/a	n/a	n/a	n/a	n/a	n/a	n/a	n/a	n/a	n/a
USA/Wyoming	Yellowstone2	2	44.7567300	-110.7377820	Duke, Nr. 43170, 43199	n/a	n/a	n/a	n/a	n/a	n/a	n/a	n/a	n/a	n/a	n/a	n/a
USA/Wyoming	USA/Wyoming1	1	44.9465760	-109.5007470	Duke, Nr. 6801	n/a	n/a	n/a	n/a	n/a	n/a	n/a	n/a	n/a	n/a	n/a	n/a
USA/Wyoming	USA/Wyoming2	1	44.9121830	-109.5204020	Duke, Nr. 6871	n/a	n/a	n/a	n/a	n/a	n/a	n/a	n/a	n/a	n/a	n/a	n/a
USA/Wyoming	USA/Wyoming4	1	44.9177140	-109.4818640	Duke, Nr. 7704	n/a	n/a	n/a	n/a	n/a	n/a	n/a	n/a	n/a	n/a	n/a	n/a
USA/Wyoming	USA/Wyoming4	1	44.9177140	-109.4818640	Duke, Nr. 7706	n/a	n/a	n/a	n/a	n/a	n/a	n/a	n/a	n/a	n/a	n/a	n/a
USA/Wyoming	USA/Wyoming5	1	44.9206920	-109.5015980	Duke, Nr. 7781	n/a	n/a	n/a	n/a	n/a	n/a	n/a	n/a	n/a	n/a	n/a	n/a
USA/Wyoming	USA/Wyoming6	1	44.9502210	-109.4834090	Duke, Nr. 8251	n/a	n/a	n/a	n/a	n/a	n/a	n/a	n/a	n/a	n/a	n/a	n/a
USA/Wyoming	USA/Wyoming7	1	44.9400760	-109.5089360	Duke, Nr. 8300	n/a	n/a	n/a	n/a	n/a	n/a	n/a	n/a	n/a	n/a	n/a	n/a
USA/Wyoming	USA/Wyoming3	1	44.9112667	-109.5187000	Duke, Nr. 6900	n/a	n/a	n/a	n/a	n/a	n/a	n/a	n/a	n/a	n/a	n/a	n/a

			Microsatellite alelles										
			22	10	18	1	9	30	19	14	17	20	29
Isolation number	Locality name	Nr. of alleles per loci	5	19	7	3	15	6	9	23	5	6	7
EM_328	Central Rhodopes – Near the jazovir Beglika		102	244	135	248	172	140	261	189	155	290	201
EM_329	Central Rhodopes – Near the jazovir Beglika		102	244	135	248	172	140	261	189	155	290	201
EM_330	Central Rhodopes – Near the jazovir Beglika		102	244	135	248	172	140	261	189	155	290	201
EM_331	Central Rhodopes – Near the jazovir Beglika		102	244	135	248	172	140	261	189	155	290	201
EM_332	Central Rhodopes – Near the jazovir Beglika		102	244	135	248	172	140	261	189	155	290	201
EM_378	Pirin – Izvorite		102	240	138	248	197	140	272	219	155	286	198
EM_379	Pirin – Izvorite		102	240	138	248	0	140	272	219	155	286	201
EM_293	Pirin – NW from Bezbog, Disilishko reka		99	240	129	248	172	140	268	241	0	0	0
EM_294	Pirin – NW from Bezbog, Disilishko reka		99	240	129	248	172	140	268	241	0	0	0
EM_295	Pirin – NW from Bezbog, Disilishko reka		99	240	129	248	172	140	268	243	155	290	201
EM_296	Pirin – NW from Bezbog, Disilishko reka		99	240	129	248	172	140	268	243	155	290	201
EM_297	Pirin – NW from Bezbog, Disilishko reka		99	240	129	248	172	140	268	249	155	290	201
EM_298	Pirin, Golyamo Valyavishko ozero		102	235	144	248	172	140	268	189	155	290	201
EM_299	Pirin, Golyamo Valyavishko ozero		102	235	144	248	172	140	268	189	155	290	201
EM_300	Pirin, Golyamo Valyavishko ozero		102	235	0	0	0	140	268	0	155	290	201
EM_301	Pirin, Golyamo Valyavishko ozero		102	235	0	0	0	0	0	0	155	290	201
EM_302	Pirin, Golyamo Valyavishko ozero		102	235	0	0	0	140	268	0	155	290	201
EM_359	Rila – Govedartsi		99	242	132	248	172	140	272	207	157	286	201
EM_360	Rila – Govedartsi		99	242	132	248	172	140	272	207	157	286	201
EM_361	Rila – Govedartsi		99	242	132	248	172	140	272	207	157	286	201
EM_376	Rila – Kazanishka reka (Kostenecki Balkan)		102	242	144	248	176	140	272	221	155	290	201
EM_377	Rila – Kazanishka reka (Kostenecki Balkan)		102	242	144	248	176	140	272	221	155	290	201
EM_303	Rila – Malyovitsa		102	244	138	248	176	140	272	217	0	0	0
EM_304	Rila – Malyovitsa		102	244	138	248	176	140	272	217	155	290	201
EM_305	Rila – Malyovitsa		102	244	138	248	176	140	272	217	155	290	201
EM_306	Rila – Malyovitsa		102	244	0	0	0	140	272	217	155	290	201
EM_307	Rila – Malyovitsa		102	244	0	0	0	140	272	217	155	290	201
EM_289	Rila – Sedemse Ozera		102	244	0	0	0	140	272	219	155	290	201
EM_290	Rila – Sedemse Ozera		102	244	0	0	0	140	272	219	155	290	201
EM_291	Rila – Sedemse Ozera		102	244	0	0	0	140	272	219	0	0	0
EM_292	Rila – Sedemse Ozera		102	244	138	248	176	140	272	219	155	290	201
EM_308	Stara Planina – Botev, Kafadikildi		102	225	144	248	172	140	272	209	155	293	201
EM_309	Stara Planina – Botev, Kafadikildi		102	225	144	248	172	140	272	209	155	293	201
EM_310	Stara Planina – Botev, Kafadikildi		102	225	144	248	172	140	272	209	155	293	201
EM_311	Stara Planina – Botev, Kafadikildi		102	225	144	248	172	140	272	209	155	293	201
EM_312	Stara Planina – Botev, Kafadikildi		102	225	144	248	172	140	272	209	0	0	0
EM_313	Stara Planina – Vartopa (pod Veženem)		102	240	144	248	172	140	272	209	155	290	201
EM_314	Stara Planina – Vartopa (pod Veženem)		102	240	144	248	172	140	272	209	155	290	201
EM_315	Stara Planina – Vartopa (pod Veženem)		102	240	144	248	172	140	272	209	155	290	201
EM_316	Stara Planina – Vartopa (pod Veženem)		102	240	144	248	172	140	272	209	155	290	201
EM_317	Stara Planina – Vartopa (pod Veženem)		102	240	144	248	172	140	272	209	155	290	201
EM_368	Vitosha - between Aleko and Platoto		102	244	0	248	178	140	272	239	155	290	201
EM_369	Vitosha - between Aleko and Platoto		102	244	0	248	178	140	272	245	0	0	0
EM_370	Vitosha - between Aleko and Platoto		102	244	0	248	178	140	272	245	157	290	201
EM_374	Vitosha – 3km S of Zlatni Mostove		102	240	132	248	174	140	275	249	155	290	201
EM_375	Vitosha – 3km S of Zlatni Mostove		102	240	132	248	174	140	275	247	155	290	201
EM_371	Vitosha – above Zvjezditsa challet		102	240	132	248	174	140	275	189	155	290	201
EM_372	Vitosha – above Zvjezditsa challet		102	240	0	0	0	140	275	189	155	290	201
EM_373	Vitosha – above Zvjezditsa challet		102	240	132	248	174	140	275	189	155	290	201
EM_365	Vitosha – Kapaklivec		102	244	0	248	178	140	272	245	157	290	201
EM_366	Vitosha – Kapaklivec		102	244	0	248	178	140	272	245	157	290	201
EM_367	Vitosha – Kapaklivec		102	244	135	248	178	140	272	245	157	290	201
EM_382	Vitoska – between Sredetsa and Lavcheto		102	247	0	248	178	140	272	249	157	290	201
EM_383	Vitoska – between Sredetsa and Lavcheto		102	247	0	248	178	140	272	249	157	290	201
EM_318	Vitoska – Cherni Vrah		102	240	144	248	172	140	275	189	155	290	201
EM_319	Vitoska – Cherni Vrah		102	240	0	0	0	0	0	0	155	290	201
EM_320	Vitoska – Cherni Vrah		102	240	0	0	0	0	0	0	155	290	201
EM_321	Vitoska – Cherni Vrah		102	240	144	248	172	140	275	189	155	290	201
EM_322	Vitoska – Cherni Vrah		102	240	144	248	172	140	275	189	155	290	201
EM_174	Borová: Pod Koupalištěm		99	240	129	248	176	140	268	189	155	290	201
EM_175	Borová: Pod Koupalištěm		99	240	129	248	176	140	268	189	155	290	201
EM_176	Borová: Pod Koupalištěm		99	240	129	248	176	140	268	189	155	290	201
EM_144	Borova: Pod nadrazim		102	240	138	248	174	140	268	239	155	290	201
EM_145	Borova: Pod nadrazim		102	240	138	248	174	140	268	233	155	290	201
EM_146	Borova: Pod nadrazim		102	240	138	248	174	140	268	239	155	290	201
EM_156	Buchtovka		102	244	132	248	172	140	272	235	155	290	201
EM_157	Buchtovka		102	244	132	248	172	140	272	235	155	290	201
EM_158	Buchtovka		102	244	132	248	172	140	272	235	155	290	201
EM_041	Častá		102	225	144	248	172	140	264	189	155	290	201
EM_042	Častá		102	225	144	248	172	0	0	0	0	290	201
EM_043	Častá		102	225	144	248	172	140	264	189	155	290	201
EM_044	Častá		102	225	144	248	172	0	0	0	0	290	201
EM_045	Častá		102	225	144	248	172	140	264	189	155	290	201
EM_251	Častá		102	225	144	248	172	140	264	189	155	290	201
EM_252	Častá		102	225	144	248	172	140	264	189	155	290	201
EM_253	Častá		102	225	144	248	172	140	264	189	155	290	201
EM_183	Čermákovy louky		102	223	132	248	174	140	268	207	155	290	201
EM_184	Čermákovy louky		102	223	132	248	174	140	268	207	155	290	201
EM_185	Čermákovy louky		102	223	132	248	174	140	268	207	155	290	201
EM_171	Damašek		99	239	138	248	172	140	268	209	155	290	201
EM_172	Damašek		99	239	138	248	172	140	268	209	155	290	201
EM_173	Damašek		99	239	138	248	172	140	268	209	155	290	201
EM_216	Filipova Hut		102	240	132	248	174	140	268	189	155	290	201
EM_217	Filipova Hut		102	240	132	248	174	140	268	189	155	290	201
EM_218	Filipova Hut		102	240	132	248	174	140	268	189	155	290	201
EM_071	Chvojnov		102	235	132	248	172	140	272	189	155	290	201
EM_072	Chvojnov		102	235	132	248	172	140	272	189	155	290	201
EM_074	Chvojnov		102	235	132	248	172	140	272	189	155	290	201
EM_075	Chvojnov		102	235	132	248	172	140	272	189	155	290	201
EM_267	Chvojnov		102	235	132	248	172	140	272	189	155	290	201

		22	10	18	1	9	30	19	14	17	20	29
EM_268	Chvojnov	102	235	132	248	172	140	272	189	155	290	201
EM_269	Chvojnov	102	235	132	248	172	140	272	189	155	290	201
EM_187	Kaliště 2A	99	225	129	248	172	140	272	189	155	290	201
EM_285	Kaliště 2A	99	225	129	248	172	140	272	189	155	290	201
EM_286	Kaliště 2A	99	225	129	248	172	140	272	189	155	290	201
EM_189	Kaliště 2B	102	242	129	248	172	140	268	189	155	290	201
EM_190	Kaliště 2B	102	242	129	248	172	140	268	189	155	290	201
EM_191	Kaliště 2B	102	242	129	248	172	140	268	189	155	290	201
EM_141	Kvilda	99	240	0	248	172	140	268	189	155	293	201
EM_142	Kvilda	99	242	157	248	172	140	272	189	155	290	201
EM_143	Kvilda	99	242	157	248	172	140	272	189	155	290	201
EM_195	Louky u Černého lesa	102	223	132	248	174	140	268	207	155	293	201
EM_196	Louky u Černého lesa	102	223	132	248	174	140	268	207	155	293	201
EM_197	Louky u Černého lesa	102	223	132	248	174	140	268	207	155	293	201
EM_201	Louky u Jeníkova	99	240	129	248	176	140	268	189	155	290	201
EM_202	Louky u Jeníkova	99	240	129	248	176	140	268	189	155	290	201
EM_203	Louky u Jeníkova	99	240	129	248	176	140	268	189	155	290	201
EM_348	Malá Kotlina9	99	235	129	248	172	140	261	233	155	290	201
EM_349	Malá Kotlina9	99	235	129	248	172	140	261	233	155	290	201
EM_350	Malá Kotlina9	99	235	129	248	172	140	261	233	155	290	201
EM_351	Malá Kotlina9	99	235	129	248	172	140	261	233	155	290	201
EM_352	Malá Kotlina9	99	235	129	248	172	140	261	233	155	290	201
EM_425	Malá Kotlina9	99	235	129	248	172	140	261	233	155	290	201
EM_065	Mechové údolí 2	0	0	129	248	185	0	0	0	0	0	0
EM_257	Mechové údolí 2	0	0	129	248	185	143	275	0	0	0	0
EM_258	Mechové údolí 2	0	0	129	248	185	0	0	0	0	0	0
EM_207	Mokřady pod Vlčkem	102	225	132	248	172	140	261	229	155	290	201
EM_208	Mokřady pod Vlčkem	102	225	0	0	0	140	261	0	155	0	201
EM_209	Mokřady pod Vlčkem	102	225	0	0	0	140	261	0	155	0	201
EM_129	Na Oklice	102	235	132	248	172	140	272	189	155	290	201
EM_130	Na Oklice	102	235	132	248	172	140	272	189	155	290	201
EM_131	Na Oklice	102	235	132	248	172	140	272	189	155	290	201
EM_192	Odranec 2 (pri ceste a domoch)	99	231	129	248	172	140	268	189	155	290	201
EM_193	Odranec 2 (pri ceste a domoch)	99	231	0	0	0	140	268	189	155	290	201
EM_194	Odranec 2 (pri ceste a domoch)	99	237	0	0	0	140	272	203	155	290	201
EM_076	Plíčky	102	237	144	248	195	140	275	207	155	290	201
EM_077	Plíčky	102	237	144	248	195	140	275	207	155	290	201
EM_078	Plíčky	102	237	144	248	195	140	275	207	155	290	201
EM_079	Plíčky	102	237	144	248	195	140	275	207	155	290	201
EM_080	Plíčky	102	237	144	248	195	140	275	207	155	290	201
EM_056	Ratajské rybníky	99	240	138	248	174	140	261	189	155	290	201
EM_057	Ratajské rybníky	99	240	138	248	174	140	261	189	155	290	201
EM_058	Ratajské rybníky	99	240	138	248	174	140	261	189	155	290	201
EM_059	Ratajské rybníky	99	240	138	248	174	140	261	189	155	290	201
EM_060	Ratajské rybníky	99	240	138	248	174	140	261	189	155	290	201
EM_046	Řeka2	102	240	132	248	172	140	261	189	155	290	201
EM_047	Řeka2	102	240	132	248	172	140	261	189	155	290	201
EM_048	Řeka2	102	240	132	248	172	140	261	189	155	290	201
EM_049	Řeka2	102	240	132	248	172	140	261	189	155	290	201
EM_050	Řeka2	102	240	132	248	172	140	261	189	155	290	201
EM_108	Suchdolské rašeliniště	102	242	129	248	174	140	268	189	155	290	201
EM_109	Suchdolské rašeliniště	99	244	141	248	172	140	268	189	155	290	201
EM_110	Suchdolské rašeliniště	102	242	129	248	174	140	268	189	155	290	201
EM_165	Suchopýrek	99	225	132	248	174	140	268	221	155	290	201
EM_166	Suchopýrek	99	225	132	248	174	140	268	221	155	290	201
EM_167	Suchopýrek	99	225	132	248	174	140	268	221	155	290	201
EM_198	Šimanovské rašeliniště	99	240	132	248	172	140	268	189	155	290	201
EM_199	Šimanovské rašeliniště	99	240	132	248	172	140	268	189	155	290	201
EM_200	Šimanovské rašeliniště	99	240	132	248	172	140	268	189	155	290	201
EM_051	Švábov	99	225	129	248	172	140	272	189	155	290	201
EM_053	Švábov	99	225	129	248	172	140	272	189	155	290	201
EM_054	Švábov	99	225	129	248	172	140	272	189	155	290	201
EM_055	Švábov	99	225	129	248	172	140	272	189	155	290	201
EM_254	Švábov	99	225	129	248	172	140	272	189	155	290	201
EM_255	Švábov	99	225	129	248	172	140	272	189	155	290	201
EM_256	Švábov	99	225	129	248	172	140	272	189	155	290	201
EM_168	Trhová Kamenice2	102	240	144	248	172	140	268	189	155	290	201
EM_169	Trhová Kamenice2	102	240	144	248	172	140	268	189	155	290	201
EM_170	Trhová Kamenice2	102	242	132	248	172	140	272	189	155	290	201
EM_117	V Lísovech	102	240	132	248	172	140	272	237	155	290	201
EM_118	V Lísovech	102	240	132	248	172	140	272	237	155	290	201
EM_119	V Lísovech	102	240	132	248	172	140	272	237	155	290	201
EM_180	V Rájích	102	240	138	248	172	140	272	239	155	290	201
EM_181	V Rájích	102	240	138	248	172	140	272	239	155	290	201
EM_182	V Rájích	102	240	138	248	172	140	272	239	155	290	201
EM_356	Velká kotlina1	99	225	144	248	172	140	261	235	155	290	201
EM_357	Velká kotlina1	99	225	0	0	0	140	261	235	155	290	201
EM_358	Velká kotlina1	99	225	0	248	172	140	261	235	155	290	201
EM_430	Velká kotlina1	99	225	144	248	172	140	261	235	155	290	201
EM_353	Velká kotlina2	99	240	132	248	172	140	272	189	155	290	201
EM_354	Velká kotlina2	99	240	0	0	0	140	272	189	155	290	201
EM_355	Velká kotlina2	99	240	132	248	172	140	272	189	155	290	201
EM_333	Velká kotlina3	99	240	0	0	0	140	268	0	0	290	201
EM_334	Velká kotlina3	99	240	0	0	0	140	268	0	0	290	201
EM_335	Velká kotlina3	99	240	0	0	0	140	268	0	0	290	201
EM_336	Velká kotlina3	99	240	144	248	172	140	268	189	155	290	201
EM_337	Velká kotlina3	99	240	144	248	172	140	268	189	155	290	201
EM_423	Velká kotlina3	99	240	141	248	172	140	268	189	155	290	201
EM_162	Vortová: Návesník	102	242	144	248	174	140	261	209	155	290	201
EM_163	Vortová: Návesník	102	242	144	248	174	140	261	209	155	290	201
EM_164	Vortová: Návesník	102	242	144	248	174	140	261	209	155	290	201

		22	10	18	1	9	30	19	14	17	20	29
EM_204	Zlámanec	102	242	144	248	174	140	261	209	155	290	201
EM_205	Zlámanec	102	242	138	248	174	140	261	209	155	290	201
EM_206	Zlámanec	102	242	144	248	174	140	261	209	155	290	201
EM_159	Zlatá louka	99	240	135	248	172	140	268	235	155	290	201
EM_160	Zlatá louka	102	244	129	248	174	140	272	189	155	290	201
EM_161	Zlatá louka	99	240	135	248	172	140	268	235	155	290	201
EM_099	Žemlička	99	240	138	248	172	140	272	203	155	290	201
EM_100	Žemlička	99	240	0	0	0	140	272	203	155	290	201
EM_101	Žemlička	99	240	138	248	172	140	272	203	155	290	201
EM_225	Gerlachov	99	235	144	248	172	140	272	189	155	290	201
EM_226	Gerlachov	102	242	144	248	174	140	272	189	155	290	201
EM_227	Gerlachov	102	242	144	248	174	140	272	189	155	290	201
EM_135	Havraník (Zlatno)	102	240	129	248	172	140	268	189	155	290	201
EM_136	Havraník (Zlatno)	102	225	138	248	172	140	268	231	155	290	201
EM_137	Havraník (Zlatno)	102	225	138	248	172	140	268	231	0	0	0
EM_279	Havraník (Zlatno)	102	225	138	248	172	140	268	231	155	290	201
EM_280	Havraník (Zlatno)	102	225	129	248	172	140	268	231	155	290	201
EM_281	Havraník (Zlatno)	102	225	0	0	0	140	268	231	155	290	201
EM_032	Hnilecká jelšina (Dobš. ľad. jaskyňa)	99	240	129	248	174	140	268	189	155	290	201
EM_033	Hnilecká jelšina (Dobš. ľad. jaskyňa)	99	240	129	248	174	140	268	189	155	290	201
EM_034	Hnilecká jelšina (Dobš. ľad. jaskyňa)	99	240	129	248	174	140	268	189	155	290	201
EM_035	Hnilecká jelšina (Dobš. ľad. jaskyňa)	99	240	129	248	174	140	268	189	155	290	201
EM_245	Hnilecká jelšina (Dobš. ľad. jaskyňa)	99	240	132	248	172	0	0	0	155	290	201
EM_246	Hnilecká jelšina (Dobš. ľad. jaskyňa)	99	240	132	248	172	140	272	219	155	290	201
EM_247	Hnilecká jelšina (Dobš. ľad. jaskyňa)	99	240	132	248	172	140	272	219	155	290	201
EM_104	Hnilecká jelšina (Pusté pole)	99	240	132	248	172	140	272	219	155	290	201
EM_037	Jalovec	102	240	132	248	172	140	268	237	155	290	201
EM_038	Jalovec	102	240	132	248	172	140	268	237	155	290	201
EM_039	Jalovec	102	240	132	248	172	140	268	237	155	290	201
EM_040	Jalovec	102	240	132	248	172	140	268	237	155	290	201
EM_011	Jochy	102	240	138	248	174	140	261	189	155	290	201
EM_012	Jochy	102	240	138	248	174	140	261	189	155	290	201
EM_013	Jochy	102	240	138	248	174	140	261	189	155	290	201
EM_014	Jochy	102	240	138	248	174	140	261	189	155	290	201
EM_015	Jochy	99	240	129	248	173	140	268	237	155	290	201
EM_123	Liptovská Lužná 1	102	225	132	248	172	140	268	189	155	290	201
EM_124	Liptovská Lužná 1	102	225	132	248	172	140	268	189	155	290	201
EM_273	Liptovská Lužná 1	102	242	144	248	172	0	0	0	0	0	0
EM_274	Liptovská Lužná 1	102	242	132	248	172	140	268	207	155	290	201
EM_274	Liptovská Lužná 1	102	242	132	248	172	140	268	207	155	290	201
EM_275	Liptovská Lužná 1	102	242	132	248	172	0	0	0	0	0	0
EM_126	Liptovská Lužná 2	99	235	129	248	195	140	261	221	155	290	201
EM_127	Liptovská Lužná 2	99	235	129	248	195	140	261	221	155	290	201
EM_128	Liptovská Lužná 2	0	0	129	248	195	0	0	0	0	0	0
EM_276	Liptovská Lužná 2	0	0	0	0	0	140	275	191	0	293	201
EM_277	Liptovská Lužná 2	0	0	129	248	195	140	272	191	155	290	201
EM_278	Liptovská Lužná 2	0	0	129	248	195	140	275	191	155	290	201
EM_219	Liptovská Lužná 3	99	235	129	248	195	140	261	221	155	290	201
EM_220	Liptovská Lužná 3	99	235	129	248	195	140	261	221	155	290	201
EM_221	Liptovská Lužná 3	99	235	129	248	195	140	261	221	155	290	201
EM_026	Liptovská Teplička 1	102	240	129	248	172	140	275	189	155	290	201
EM_027	Liptovská Teplička 1	102	240	129	248	172	140	275	189	155	290	201
EM_028	Liptovská Teplička 1	102	240	129	248	172	140	275	189	155	290	201
EM_029	Liptovská Teplička 1	102	240	129	248	172	140	275	189	155	290	201
EM_030	Liptovská Teplička 1	102	240	129	248	172	140	275	189	155	290	201
EM_138	Liptovská Teplička 1	99	242	144	248	172	140	272	235	155	290	201
EM_139	Liptovská Teplička 1	99	242	144	248	172	140	272	235	155	290	201
EM_283	Liptovská Teplička 1	102	240	129	248	172	140	275	189	155	290	201
EM_284	Liptovská Teplička 1	102	240	129	248	172	140	275	189	155	290	201
EM_132	Liptovská Teplička 2	99	235	129	248	172	140	275	239	155	290	198
EM_133	Liptovská Teplička 2	102	225	132	248	174	140	272	189	155	286	201
EM_134	Liptovská Teplička 2	99	235	129	248	172	140	275	239	155	290	198
EM_240	Liptovská Teplička 2	99	235	132	248	172	140	0	209	155	290	201
EM_241	Liptovská Teplička 2	99	235	132	248	172	0	0	0	0	0	0
EM_228	Podbanské, pri chate u K. 1	102	240	144	248	178	140	275	189	155	290	201
EM_229	Podbanské, pri chate u K. 1	99	227	138	248	195	140	268	189	155	290	201
EM_230	Podbanské, pri chate u K. 1	102	240	144	248	178	140	275	189	155	290	201
EM_096	Pribylina - Pedicularis s.-c.	102	244	129	261	185	140	268	241	155	290	201
EM_097	Pribylina - Pedicularis s.-c.	102	244	144	248	174	140	268	239	155	290	201
EM_098	Pribylina - Pedicularis s.-c.	102	242	132	248	172	140	268	207	155	290	201
EM_114	Pribylina - za Esperantem	102	237	132	248	172	140	272	221	155	290	201
EM_115	Pribylina - za Esperantem	102	240	144	248	172	140	272	189	155	290	201
EM_116	Pribylina - za Esperantem	102	240	144	248	172	0	0	0	0	0	0
EM_222	Pribylina - za Esperantem	102	240	144	248	172	140	272	189	155	290	201
EM_223	Pribylina - za Esperantem	102	240	144	248	172	140	272	189	155	290	201
EM_224	Pribylina - za Esperantem	102	240	144	248	172	140	272	189	155	290	201
EM_270	Pribylina - za Esperantem	102	240	144	248	172	140	272	189	155	290	201
EM_271	Pribylina - za Esperantem	102	240	0	0	0	140	272	189	0	290	201
EM_272	Pribylina - za Esperantem	102	240	0	0	0	140	272	189	0	290	201
EM_016	Puchmajerovej jazierko (opodiaľ Kubínskej hole)	102	240	138	248	172	140	268	231	155	290	201
EM_017	Puchmajerovej jazierko (opodiaľ Kubínskej hole)	102	240	138	248	172	140	268	231	155	290	201
EM_018	Puchmajerovej jazierko (opodiaľ Kubínskej hole)	102	240	138	248	172	140	268	231	155	290	201
EM_019	Puchmajerovej jazierko (opodiaľ Kubínskej hole)	102	240	138	248	172	140	268	231	155	290	201
EM_020	Puchmajerovej jazierko (opodiaľ Kubínskej hole)	102	240	138	248	172	140	268	231	155	290	201
EM_231	Švihrová 2	99	240	129	248	172	140	268	235	155	290	201
EM_232	Švihrová 2	99	240	129	248	172	140	268	235	155	290	201
EM_233	Švihrová 2	99	240	129	248	172	140	268	235	155	290	201
EM_105	Telgárt, Pšolinec 1	102	255	132	248	174	140	272	189	155	290	201
EM_106	Telgárt, Pšolinec 1	102	255	132	248	174	140	272	189	155	290	201
EM_107	Telgárt, Pšolinec 1	102	255	132	248	174	140	272	189	155	290	201
ST_1313	Alberta	106	244	141	248	187	136	258	0	161	279	198
EM_400	Canada/British columbia2	106	247	135	248	167	147	275	197	152	290	195
EM_401	Canada/British columbia2	102	242	129	248	172	140	275	207	155	290	201

		22	10	18	1	9	30	19	14	17	20	29
EM_421	Canada/British columbia3	102	223	135	248	199	140	268	235	155	293	201
EM_398	Canada/New Brunswick	106	230	129	248	0	143	266	197	0	0	0
EM_399	Canada/New Foundland	102	244	129	248	172	140	268	213	157	290	201
EM_396	Canada/Quebec	0	0	132	248	165	136	266	203	0	286	201
EM_395	Canada/Yukon	102	240	138	248	172	140	264	233	155	286	201
EM_388	Finland1	99	223	129	248	172	140	268	189	155	290	201
EM_387	Finland2	102	237	138	248	197	140	261	189	155	290	201
ST_580	Finland3	102	225	144	248	172	140	264	207	155	284	201
EM_391	Japan	106	231	132	248	0	143	266	235	155	279	194
ST_1310	Luxembourg	99	227	132	243	183	123	261	189	152	279	194
EM_390	Norway2	102	233	132	248	185	127	258	197	159	290	198
ST_71	Trondelag	102	225	138	248	172	140	264	189	157	290	201
EM_120	Rospuda basin, Szczebecka	99	240	129	248	172	140	272	189	155	290	201
EM_121	Rospuda basin, Szczebecka	99	240	129	248	172	140	272	189	0	290	0
EM_122	Rospuda basin, Szczebecka	99	240	129	248	172	140	272	189	155	290	0
EM_392	Pologne	86	259	132	248	185	140	255	193	155	293	194
ST_73	Ayon Island	109	247	138	248	185	147	272	249	155	293	210
ST_74	Wrangel Island1	109	261	132	248	180	147	266	247	157	293	222
ST_75	Wrangel Island2	109	259	132	248	180	147	0	247	0	293	222
EM_393	Spain	99	242	135	248	172	140	268	189	155	290	201
ST_72	Jamtlad	102	237	138	248	172	140	261	231	155	284	201
EM_389	Sweden1	102	223	144	248	178	140	275	189	155	290	201
EM_384	Switzerland	102	253	138	248	197	140	268	189	155	290	201
ST_70	Berwickshire	102	223	129	248	172	140	275	189	155	290	201
ST_371	USA/Alaska	109	257	135	248	0	140	0	221	159	284	201
EM_420	USA/Alaska	99	225	144	248	172	140	275	189	155	290	201
EM_410	USA/Connecticut	102	235	129	248	172	140	268	207	155	293	204
EM_411	USA/Idaho	102	240	129	248	172	140	268	229	155	295	201
ST_1312	USA/Maine	102	235	129	248	172	140	268	233	155	290	201
EM_404	USA/Maine	99	242	138	248	172	127	275	209	155	290	201
ST_1311	USA/Michigan	99	240	129	248	172	140	264	189	155	293	201
EM_405	USA/Minnessota	102	0	138	248	199	140	268	233	155	290	201
EM_406	USA/New Hampshire	99	227	138	248	172	140	268	215	155	286	198
EM_407	USA/New Jersey	99	240	138	248	172	140	275	189	155	290	201
EM_408	USA/Oregon	102	242	129	248	172	140	268	207	155	290	198
ST_1322	USA/South Carolina	0	0	138	0	169	136	266	191	0	284	198
EM_402	USA/Utah	102	225	129	248	0	127	268	235	155	293	201
EM_403	USA/Vermont	102	240	129	248	172	127	268	219	155	290	201
EM_429	USA/Wyoming/Yellowstone2	102	242	138	248	172	140	275	221	155	293	201
EM_418	USA/Wyoming1	99	220	129	248	172	140	275	207	155	290	201
EM_412	USA/Wyoming2	99	220	129	248	172	140	275	207	155	290	201
EM_413	USA/Wyoming3	99	220	129	248	172	140	275	207	0	290	0
EM_416	USA/Wyoming4	99	220	129	248	172	140	275	207	155	290	201
EM_417	USA/Wyoming4	99	220	129	248	172	140	275	207	155	290	201
EM_419	USA/Wyoming5	99	220	129	248	172	140	275	207	155	290	201
EM_414	USA/Wyoming6	99	235	129	248	172	140	278	219	155	290	201
EM_415	USA/Wyoming7	99	220	129	248	172	140	275	207	155	290	201
